# Male Reproductive Traits Display Increased Phenotypic Variation in Response to Resource Quality and Parental Provisioning in a Tropical Rainforest Dung Beetle, *Onthophagus* c.f. *babirussa*

**DOI:** 10.1002/ece3.70421

**Published:** 2024-10-14

**Authors:** Sean Yap, Kai Xin Toh, Nalini Puniamoorthy

**Affiliations:** ^1^ Department of Biological Sciences National University of Singapore Singapore

**Keywords:** condition dependence, larval food quality, parental provisioning, phenotypic plasticity, reproductive evolution, sexual selection

## Abstract

Reproductive traits that mediate differential fitness associated with mate acquisition and fertilisation success are often strongly linked to the overall condition. We investigated the effects of resource quality and parental provisioning in the phenotypic expression of sexual and non‐sexual traits in a rainforest dung beetle, *Onthophagus* c.f. *babirussa* (Eschscholtz, 1822) from Singapore. F1 individuals were reared from wild‐caught beetles and paired up to produce offspring (F2), and F2 larvae from the same F1 parents were reared on two dung substrates (herbivore and omnivore) in a full‐sib design. Sexual traits displayed greater phenotypic variation in response to dung resource quality, with the precopulatory trait (horn length) responding more than the postcopulatory trait (testes weight). Notably, genotype‐by‐environment interactions between parental lines (genotype) and dung type (environment) affected male body size and horn length only, suggesting sex‐specific variance in plasticity associated with sexually selected precopulatory traits. Dung type had significant effects on all measured traits. Offspring that were provisioned higher quality resource (omnivore dung) had larger absolute and relative trait values. Parental lines only significantly affected female body size but none of the male traits, suggesting an important role of environment *and* resource partitioning in determining precopulatory success of male offspring. Parental provisioning of larval resource varied with resource quality and brood sequence. Parents provisioned more dung when herbivore dung was presented than when they were given omnivore dung and provisioned more dung for their earlier broods when using herbivore dung but not omnivore dung. This suggests a trade‐off between early offspring fitness and resource quality. We tested directly for genotype‐by‐environment (G × E) interactions in the expression of several morphological traits relevant to dung beetle fitness and documented that offspring with similar phenotypes may result from completely different parental resource allocation strategies. We discuss the importance of studying parental investment on trait variation and its implications on dung beetle ecology.

## Graphical abstract

We investigated the effects of resource quality and parental provisioning in the phenotypic expression of sexual and non‐sexual traits in a rainforest dung beetle, Onthophagus babirussa (Eschscholtz, 1822) from Singapore. ‘Higher quality’ dung resulted in offspring exhibiting greater phenotypic fitness, and mother beetles compensated dung quality with the dung amount when using ‘lower quality’ dung. Genotype‐by‐environment interactions between parental lines (genotype) and dung type (environment) affected male body size and horn length only, suggesting sex‐specific variance in plasticity associated with sexually selected precopulatory traits.

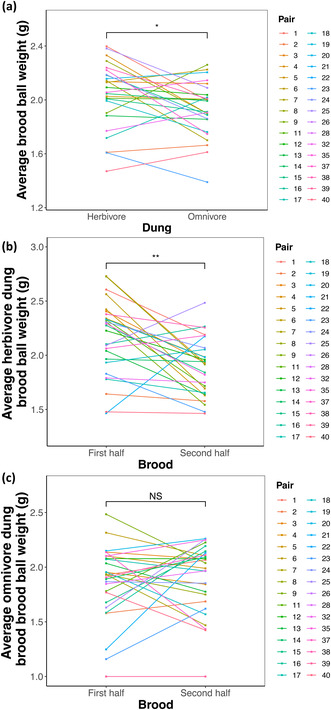

## 1 Introduction

An organism's environment has strong effects on the phenotypic variation of most traits that influence fitness. Theory predicts that optimal allocation of limited resources (condition) differs among traits, shown through differing degrees of condition‐dependent expression. For example, traits undergoing strong, directional sexual selection often evolve greater sensitivity to condition during development than non‐sexually selected structures (Bonduriansky 2007; Bonduriansky et al. 2008; House and Simmons 2007; Lorch et al. 2003; Oudin, Bonduriansky, and Rundle 2015; Rodríguez‐Muñoz et al. 2010). Even among sexually selected traits, condition dependence may vary depending on their function, for example, those involved in pre‐ versus postcopulatory sexual selection. Comparing the condition dependence of non‐sexual, pre‐ and postcopulatory sexual traits would highlight the importance of certain courtship and/or reproductive behaviours that drive evolution and speciation in a lineage. Additionally, patterns of condition dependence may be affected by resources of different origins. In species that engage in parental provisioning of offspring, condition can be affected by the nutritional quality of resources available in the surrounding environment and its relationship with the sheer quantity of resources provided that is determined by parental behaviour. For example, parents may exhibit compensatory provisioning efforts in times or spaces of poor abailable resource quality (Senécal et al. 2021). In this study, we investigated the effect of resource type on parental provisioning and condition dependence of offspring non‐sexual and pre‐ and postcopulatory sexual traits in a dung beetle.

Insects are useful model organisms in such studies, as many species display condition‐dependent sexually dimorphic structures (Esperk et al. 2007; Bonduriansky 2007; Zinna et al. 2018; Rohner and Blanckenhorn 2018; Rohner et al. 2018; Kemp and Rutowski 2007; Miller, McDonald, and Moore 2016). *Onthophagus* (Latreille, 1802) is a genus of dung beetles containing species that are model organisms in ecology and evolution, with recent studies suggesting that their morphology and genetic variation can be influenced by sexual selection, parental investment and environmental variation via a multitude of complex mechanisms (Dury, Moczek, and Schwab 2020; Hu et al. 2020; Schwab et al. 2016; Snell‐Rood et al. 2016). Males often possess thoracic and/or head horns used as weapons in male–male competition to gain access to females and defend their breeding tunnels (Cook 1987; Garcia‐Gonzalez and Simmons 2011; Kijimoto et al. 2009; Moczek et al. 2007; Simmons 2014; Simmons and García‐González 2008). These horns are diverse in shape, size and even location (Emlen et al. 2005). Horns are such an important precopulatory trait for mating success that developmental and physiological trade‐offs can exist with postcopulatory traits involved in fertilisation success, such as testes mass and sperm length (Emlen, Corley Lavine, and Ewen‐Campen 2007; McCullough, Buzatto, and Simmons 2018; Parzer and Moczek 2008; Simmons and Emlen 2006; Simmons and García‐González 2008; Simmons and Ridsdill‐Smith 2011; Simmons, Emlen, and Tomkins 2007), Often, alternative mating tactics also occur with the corresponding morphological trait variation, such as the splitting of male phenotypes into ‘major’ and ‘minor’ morphs based on body size or secondary sexual trait size where a ‘breakpoint’ developmental threshold exists. This phenomenon has been observed in multiple species of *Onthophagus*, such as *O. accuminatus* (Kotiaho and Tomkins 2001). Whether a larva falls on either side of the threshold during development is often influenced by environment and condition.

The environment and parental care are determinant for the survival and reproductive success of dung beetle offspring (Favila 1993; Halffter, Huerta, and Lopez‐Portillo 1996). Offspring's individual condition and adult phenotypes can be modulated by food type availability during larval development and affected by maternal investment through food provisioning (Emlen 1994, 1997; Moczek 1998; Servín‐Pastor et al. 2021; Silva et al. 2016), directly affecting offspring success in male–male sexual competitions (Chamorro‐Florescano, Favila, and Macías‐Ordóñez 2011; Salomão et al. 2019). Dung beetles may also display adaptive, compensatory behaviour when facing poor quality resources, such as the provisioning of greater food amounts (Servín‐Pastor et al. 2021).

Dung beetles from the genus *Onthophagus* provision food for their offspring through the construction of brood balls, balls of dung in which they deposit a single egg (Hanski and Cambefort 1991). The hatched larva spends its entire pre‐pupal development inside this brood ball, so the amount and type of dung provisioned by female beetles have important effects on the adult phenotype of their offspring. This parental effect can affect variation in male phenotypes (Arellano et al. 2015; Hunt and Simmons 2000; Kishi and Nishida 2006; Moczek 1998; Snell‐Rood et al. 2016). Food quality and amount affect plastic expression of male traits such as horn length and body size (Emlen 1994, 1997; Moczek 1998), so one would expect these traits to be condition‐dependent. Yet, previous studies on *Onthophagus taurus* that examined differences in these effects between sexual and non‐sexual traits found that secondary sexual traits (relative horn length) but not primary sexual traits (genital traits) were condition‐dependent (House and Simmons 2007). Does this hold true for other *Onthophagus* species?

In this study, we addressed two main questions: (1) How does parental provisioning influence the larval environment and to what extent does it influence development, and (2) do precopulatory, postcopulatory and non‐sexual traits respond equally to environmental variation such as variation in resource quality and to what extent does genetic background and its interaction with resource quality as an environmental effect affect phenotypic expression? We studied *Onthophagus* c.f. *babirussa*, a widespread species across Southeast Asia that exhibits strong population‐level differences sexual dimorphism, likely in response to resource availability (Goh 2014; Kudavidanage, Qie, and Lee 2012; Priawandiputra et al. 2020; Toh et al. 2022). From our field collections, we observed that omnivore dung was more attractive to *O*. c.f. *babirussa* than herbivore dung, where traps baited with herbivore dung collected up to three individuals at most, while omnivore dung‐baited traps in the same locations trapped up to 21 individuals. Thus, in the context of the populations of this species used in our study, we used omnivore dung as a ‘higher quality’ resource and herbivore dung as the ‘lower quality’ one. Given the known occurrence of compensatory breeding behaviour in dung beetles (Hunt and Simmons 2004; Servín‐Pastor et al. 2021), we examined the presence and effect of parental provisioning as a function of resource quantity (brood ball weight) and the possible interactions with resource quality (omnivore/herbivore) on offspring traits. For example, Hunt and Simmons (2004) found that parent beetles adjust their provisioning based on dung type. Likewise, we predicted that to compensate for lower quality, parents would provision greater quantities of herbivore dung than omnivore dung when both are provided ad libitum.

Next, we tested if larval food quality (dung type) affected phenotypic variation in horn length, testes weight and body size across 40 parental lines. Based on previous studies that found heritable variation for all measured traits in other *Onthophagus* species, we predicted that genotype (parental lines) and its interactions with environmental effects would affect reproductive trait expression (Buzatto et al. 2015; Simmons and Garcia‐Gonzalez 2011; Simmons et al. 2009; Simmons and Kotiaho 2002).

## 2 Materials and Methods

### 2.1 Dung Beetle Sampling

**FIGURE 1:**
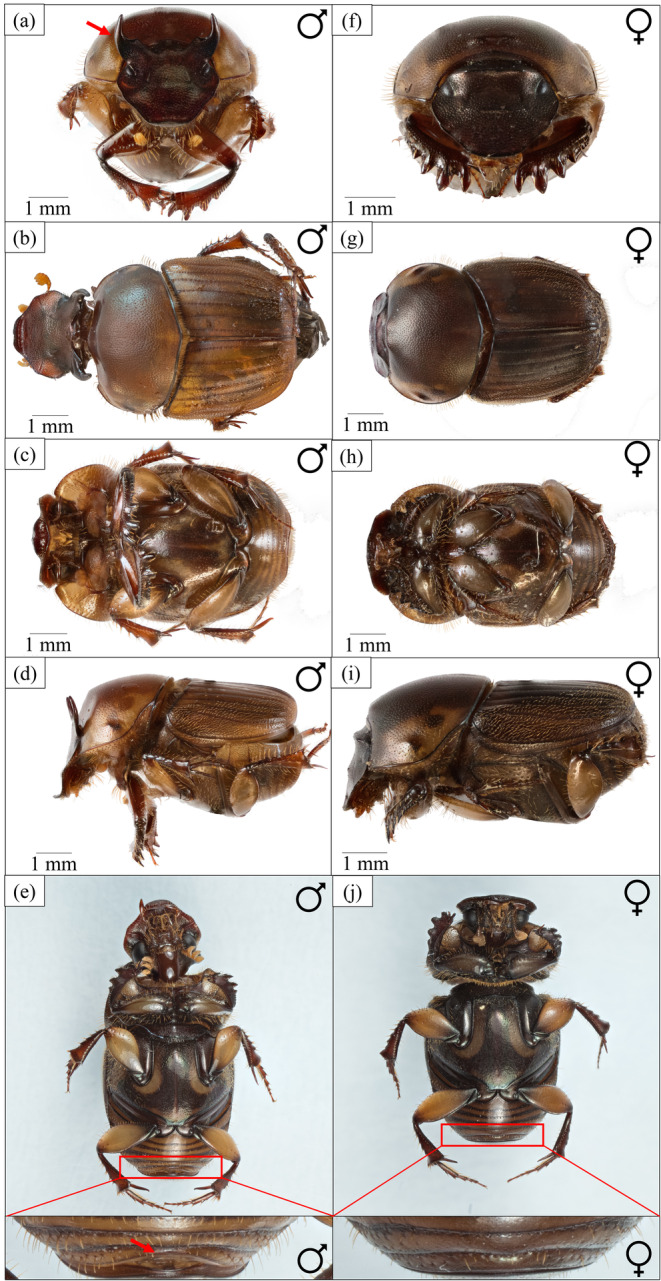
Anterior, dorsal, ventral and lateral habitus of male (a–e) and female (f–i) *O*. c.f. *babirussa*. Sexes can be distinguished via the last abdominal segment of (e) males and (j) females and the presence of head horns in males (a), as indicated by the red arrows.

Beetles were collected using dung‐baited pitfall traps and intercept traps from six sampling sites in Singapore (Table S1). Due to the close geographic proximity and the lack of genetic distinction between populations (Toh et al. 2022), individuals of the study species were pooled for this study. *Ontophagus* c.f. *babirussa* were separated from other species via distinct morphological characters, as detailed in Toh et al. (2022). Males exhibit a pair of curved head horns, the appearance and sexual dimorphism of which is depicted in Figure 1.

Sampling spanned from September 2019 to December 2019. Mixed‐sex cultures (males: *n* = 117; female: *n* = 181) were set up in the insectary located at the Department of Biological Sciences, National University of Singapore for 1 week and provided a mixture of cattle and human dung *ad libitum* in a 3:1 ratio to ensure that the beetles had mated. Females (*n* = 114) were placed in individual setups (7 × 7 × 11.5 cm plastic containers filled with 7 cm of moist lawn sand, topped dung mixture and sealed with stockings) for 1 week to construct brood balls. After 1 week, the sand was gently sifted using forceps to locate the female and her brood balls. Females were preserved in 70% ethanol, and the brood balls (*n* = 222) were incubated separately. Each emerged adult (males: *n* = 45; females: *n* = 51) was housed in individual containers and represented the first generation (F1) of beetles reared in laboratory conditions that were subsequently used for the full‐sib experiments. All beetle cultures were reared in incubators (Percival—Intellus Control System and Invictus Drosophila Incubator) with the following settings: 26°C, 78% relative humidity and a 12‐h light and 12‐h dark cycle.

## 3 Experimental Design

### 3.1 Effect of Food Quantity/Quality

One F1 male and one F1 female virgin beetle were paired in each setup (7 × 7 × 11.5 cm plastic containers filled with 7 cm of moist lawn sand). In total, 40 pairs of F1 *O*. c.f. *babirussa* were set up. For the purposes of our study, herbivore dung was used as a ‘poor’ quality while omnivore dung was used as a ‘good’ quality resource. This was based on the biological context of the study species, *O*. c.f. *babirussa*, which showed to be strongly attracted to traps baited with omnivore dung in the field, while rarely showing up in herbivore dung traps. The herbivore dung was collected from Gaur cattle (*Bos gaurus*) in the Singapore Night Safari, and the omnivore dung was contributed by members of the study (*Homo sapiens*). For half of the setups, herbivore dung was placed *ad libitum* on top of the sand. For the other half, omnivore dung was used instead. Every 3 days from first setting up, the sand was sifted to locate and collect the brood balls. If brood balls (*n* > 0) were present in a setup, they were collected and the setup was then provided dung of the alternate type before sifting again 3 days later. If brood balls were not found (*n* = 0), dung of the same type would be replaced in the setup and only changed if brood balls were detected upon sifting after another 3 days. Brood balls were collected in these 3‐day cycles until either F1 parent expired. Across the pairs, the number of brood balls for both dung types varied from 0 to 19 in total. The weight and size of the collected brood balls were measured using an electronic balance (A&D FX‐300, resolution 0.001 g) and an electronic calliper (Neiko Tools 01408A Electronic Digital Calliper, USA, resolution 0.01 mm). The brood balls were then set up individually (4.7 × 4.7 cm plastic container with a 0.5 cm layer of sand). Emerged adults (second generation, F2) were preserved in 70% ethanol. A schematic diagram of the husbandry setup is shown in Figure S1.

### 3.2 Reproductive Trait Measurements

Non‐sexual traits (male and female body size based on pronotum width) and male sexual traits (horn length and testes weight) of the F1 and F2 generation were measured in accordance with the protocol outlined by in Toh et al. (2022). Maximum pronotum width is a widely used proxy for body size because the pronotum width does not change in adulthood and has been found to be the most appropriate measure for body size in dung beetles (Emlen 1997; Knapp, Knappová, and Miller 2013). Here, the maximum pronotum width was measured using the eyepiece reticle on the Olympus SZX10 microscope (resolution 0.1 mm). Male horn length was measured following the protocol reported by Moczek and Emlen (1999) (distal tip to bottom of the outer edge of one horn). Measurements were obtained from images captured using a camera (EOS 800D and 6D camera body with the Canon MP‐E 65mm f/2.8 1‐5× lens at 5× optical zoom) suspended on the Dun, Inc. P‐51. The Camlift controller V2.9.3.0 software was used to take multiple images at different heights for focus stacking on the Zerene Stacker V. 1.04. software. The stacked image was then imported to Adobe Photoshop CS5 V. 12.0 ×64 to add a 1 mm scale bar and imported to ImageJ V. 1.51 to obtain the horn measurements. Following published protocols (Simmons and Emlen 2006), male abdomens were dissected to measure testes weight. Testes were isolated and transferred onto pre‐weighed aluminium sheets before drying in a Memmert Gravity Basic Digital Oven D overnight. The total weight was measured on the Mettler Toledo ML104 Newclassic ml Analytical Balance to a resolution of 0.00001 g.

### 3.3 Statistical Analyses

All statistical analyses were carried out in R version 3.6.3 (R Core Team 2020). The R packages *ggplot2* (Wickham 2016), *Rmisc* (Hope 2013) and *ggpubr* (Kassambara 2018) were used to construct the figures. For all results with *p*‐values, values below 0.05 were considered statistically significant.

Toh et al. (2022) found that the horn allometry of *O*. c.f. *babirussa* best fit the breakpoint model over linear, quadratic and cubic models, suggesting the presence of major and minor morphs in males. Following the same method, we first calculated static allometry by constructing scatterplots of logged horn length against logged pronotum width, and then fitted the breakpoint model using the R package segmented (Muggeo 2008). The breakpoint allometry is depicted in Figure 3. F2 males were then split into major and minor morphs based on the switchpoint for horn length, in accordance with the suggested treatment of male *Onthophagus* dung beetles in the study by Kotiaho and Tomkins (2001). Following this, models to investigate the effects of genotype‐by‐environment (G × E) interactions and the interactions between resource type and amount on the measured offspring sexual and non‐sexual traits were run on four datasets from F2 offspring—females, all males, minor males and major males.

In these analyses, *lme4* (Bates et al. 2015) was used to run linear mixed effects models for measured F2 traits as response variables, with dung type (herbivore or omnivore) and brood ball weight (amount of dung provisioned per larva) treated as the fixed environmental effects, while parental line (pair) and its interaction with dung type were treated as genetic and G × E random effects. This follows the methods to test the significance of GxE interactions on reproductive traits by Dia, Wehner, and Arellano (2017). Horn length data were square root‐transformed, and testes weight values were log‐transformed to satisfy homoscedasticity assumptions for their models, and residual plots for the models can be found in Figure S2. The other R packages used were *lmerTest* (Kuznetsova, Brockhoff, and Christensen 2017) to obtain *p*‐values and summary tables using analysis of variance (ANOVA) for fixed effects and ranova for random effects, *dplyr* (Wickham et al. 2020) for data manipulation and summarising, *afex* (Singmann, Bolker, and Westfall 2020) for ANOVA analysis of the models and *tidyr* (Wickham 2020) for data management.

To investigate the effect of larval food quality on parental provisioning, paired *t*‐test was run to determine if there were significant differences in the amount of food provisioned by parents (brood ball weight) as a function of larval food quality across the parental lines (pairs). Average brood ball weights for each dung type per parental line were recorded, and an overall *t*‐test was conducted across families. *T*‐test was used, as the data for average brood ball weight was normally distributed as determined by the Shapiro–Wilk test. The analysis was conducted on parental lines (pairs) that produced minimally five brood balls per dung type.

To investigate the effect of brood sequence on parental provisioning, a paired *t*‐test was run to determine if the amount of food provisioned by parents (brood ball weight) differed significantly between the initial and subsequent broods for each dung type provided to parental lines (pairs) to construct brood balls with. The total number of brood balls from each F1 pair were summed for each dung type and divided into two categories, initial and subsequent broods, based on brood sequence chronology. For example, for an F1 pair with six herbivore brood balls in total, the first three herbivore brood balls that were constructed would be the initial brood, while the latter three would be the subsequent brood. *T*‐test was used for herbivore dung, as the data for average herbivore brood ball weight was normally distributed using the Shapiro–Wilk test, while Wilcoxon matched‐pairs signed‐rank test was used for omnivore dung as the average omnivore brood ball weight was not normally distributed. The analysis was conducted on parental lines (pairs) that produced minimally four brood balls per dung type.

## 4 Results

### 4.1 Effect of Dung Type on Parental Provisioning

**FIGURE 2:**
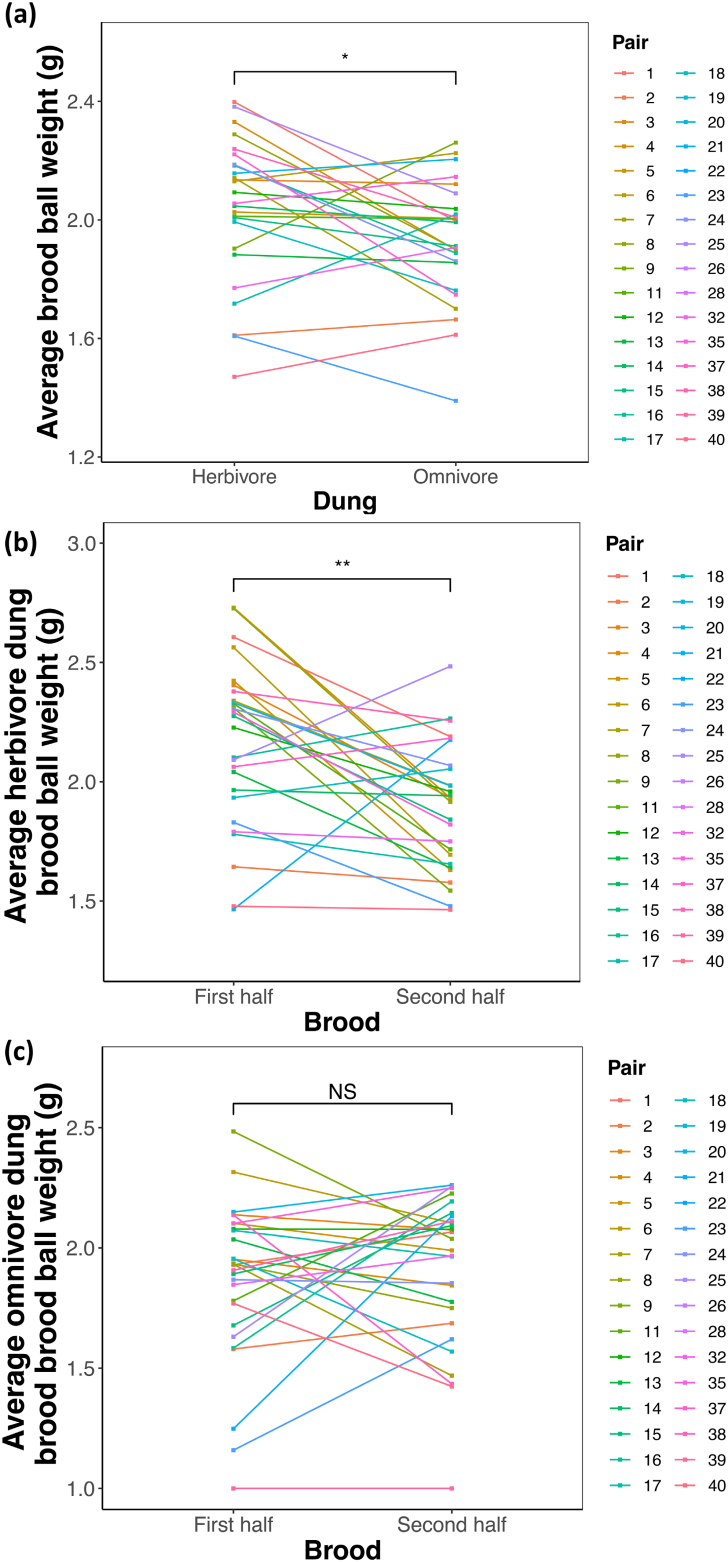
Plots illustrating (a) variation in the amount of food provisioned by parents (brood ball weight) based on food quality (dung type) (*n* = 553). (b) Variation in herbivore dung provisioned (brood ball weight) (*n* = 283) and (c) variation in omnivore dung provisioned based on the brood sequence (initial or subsequent broods) (*n* = 279). “*” = (*p* < 0.05), “**” = (*p* < 0.005), ‘NS’ = not significant, ‘*n*’ = number of brood balls.

The amount of food provisioned by parents (average brood ball weight) was significantly higher when given herbivore dung for most of the parental lines (pairs) (paired *t*‐test; *t*(25) = 2.352, *p* = 0.0268, Figure 2a). In addition, the amount of food provisioned by parents was significantly higher in the initial herbivore dung broods (paired *t*‐test; *t*[26] = 3.629, *p* = 0.0012, Figure 2b), but no significant differences were found between initial and subsequent omnivore dung broods (Wilcoxon matched‐pairs signed‐rank test; *p* = 0.6617, Figure 2c). From this, it can be inferred that brood ball weight is an important covariate, especially for herbivore dung brood balls.

From 40 F1 pairs of *Onthophagus* c.f. *babirussa*, a total of 576 brood balls were collected, of which 356 (F2) offspring emerged. Raw trait values of parents and offspring can be found in Table S3, and the analysed results are presented below. Eight pairs were excluded from analyses due to a lack of brood balls produced or lack of emerged F2 offspring.

### 4.2 Splitting of F2 Males Into Minor and Major Morphs

**FIGURE 3:**
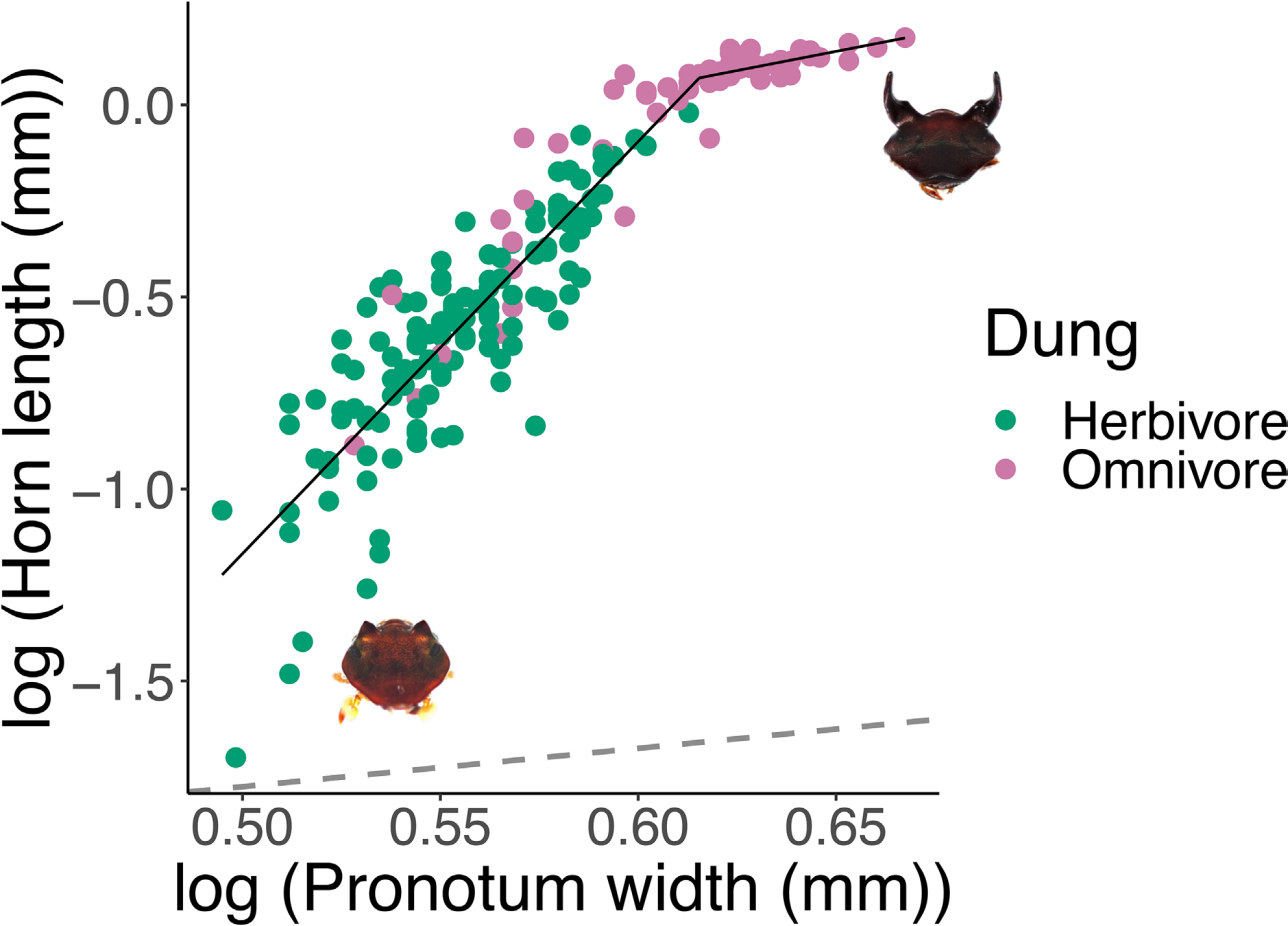
Horn length allometry fitted to the breakpoint model. The dotted grey line indicates the isometric slope with a value of 1. Appearances of major and minor male horns are depicted.

Applying the breakpoint model to the static horn allometry of F2 males, a switchpoint of 0.0675 was identified for log(horn length). Individuals with horn length values below the switchpoint were considered minor males, and those with values above the switchpoint were considered major males. This is depicted in Figure 3 below. Notably, all major males emerged from brood balls constructed with omnivore dung; thus, for subsequent analysis, major males were only modelled with brood ball weight and parental pairs as factors.

### 4.3 Effects of Genetic and Environmental Factors on Sexual and Non‐sexual Traits

From the ANOVA analyses, the interaction (G × E) between environment (dung type) and genotype (parental lines) was only significant in male body size (*p* = 0.0344) and horn length (for both overall males [*p* = 0.0023] and just minor males [*p* = 0.0033]), but not female body size or male testes weight (Table S2). Dung type (environment) significantly explained variation in female body size and male body size and horn length. Dung type had significant effects on overall male testes weight (*p* = 0.0175), but not for the subset of only minor males (Table S2). Parental lines (genotype) significantly affected female body size (*p* = 0.0371) and testes weight in only major males (*p* = 0.0349), but none of the male traits otherwise (Table S2).

**FIGURE 4:**
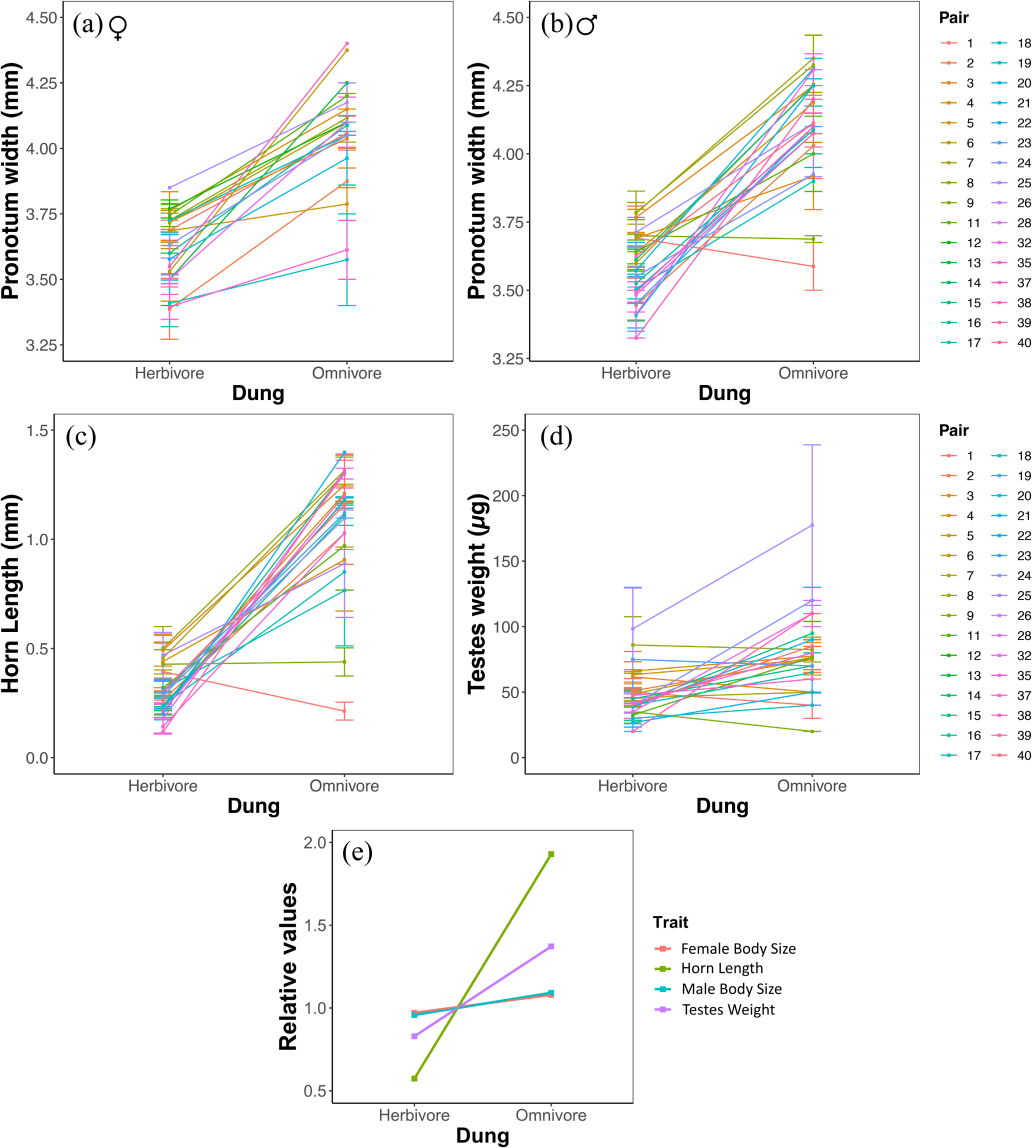
Reaction norms where lines connect average trait measurement per parental line in different dung types for (a) female (*n* = 163) body size, (b) male (*n* = 193) body size, (c) male horn length and (d) male testes weight in *O*. c.f. *babirussa* subjected to different larval food quality (dung type) and (e) slopes of relative values for the four traits divided by dung type. Lines connect average body size per parental line in different dung types. “*n*” = number of measured offspring.

Plotting the reaction norms (Figure 4a–d) and the relative values (dividing trait measurements by total trait average, then averaging these values by dung type) (Figure 4e) showed that offspring had greater absolute and relative trait values when raised on omnivore dung than on herbivore dung. Comparing the relative slopes (Figure 4e) also shows that dung type has a stronger effect on horn length (precopulatory sexual trait) and testes weight (postcopulatory sexual trait) as compared to pronotum width (non‐sexual trait). Dung type also had a greater effect on phenotypic variation in horn length than testes weight.

**FIGURE 5:**
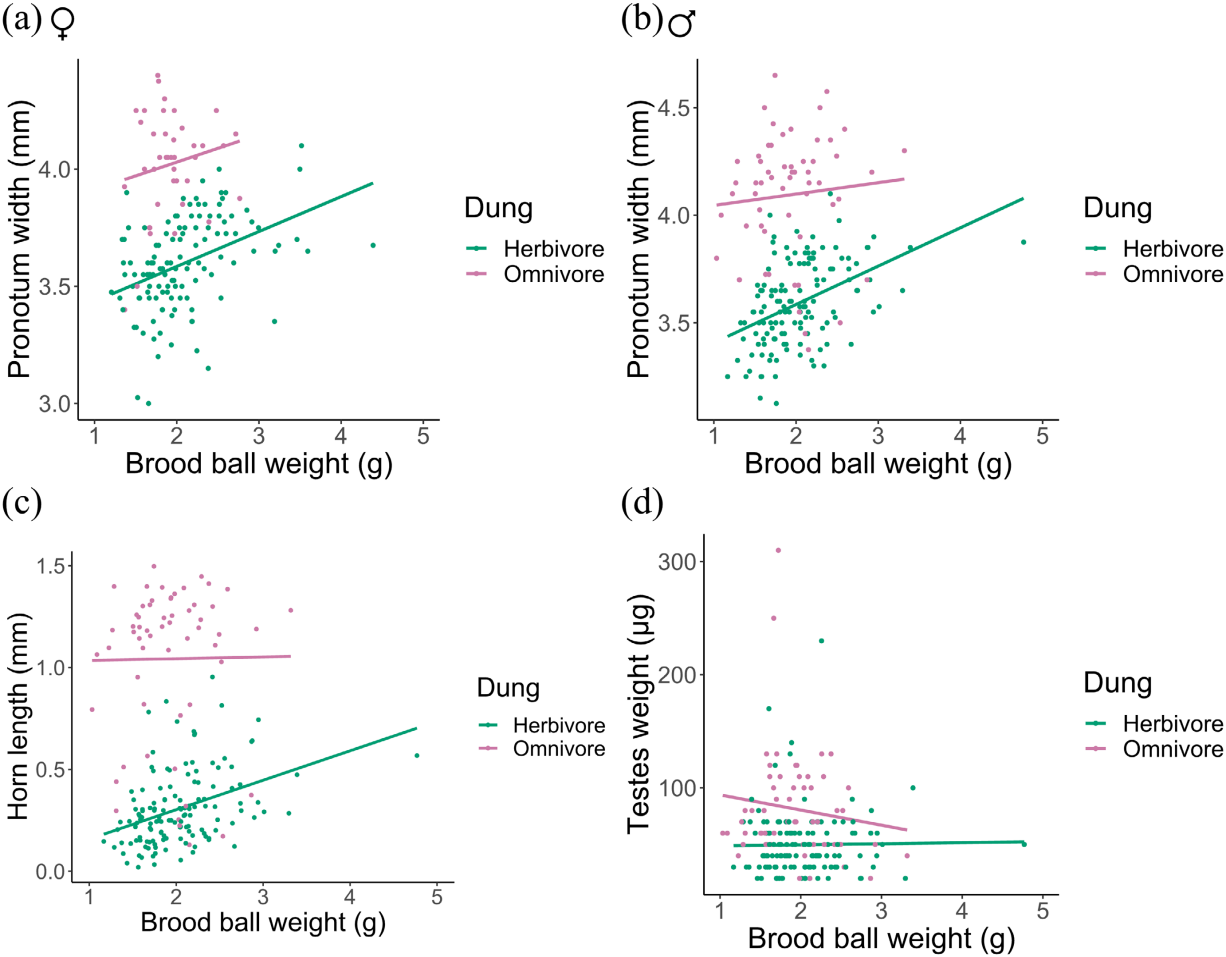
Effects of larval food quality (dung type) and amount of food provisioned (brood ball weight) on (a) body size (pronotum width) in female, (b) body size (c) horn length and (d) testes weight in male *O*. c.f. *babirussa*.

The ANOVA also showed that the amount of food provisioned (brood ball weight) significantly affected body size for both females (*p* = 0.0343) and overall males (*p* = 0.0094), but not the minor and major male subsets (Table S2). Both female (Figure 5a) and overall male (Figure 5b) offspring showed larger body sizes when raised on omnivore dung compared to herbivore dung, and body size increased with brood ball weight. The interaction between the amount of food provisioned and quality (dung type) did not affect body size for males and females (Table S2). The amount of food provisioned and its interaction with food quality significantly affected horn length for males overall (*p* = 0.0483) (Table S2), where horn length was greater in offspring provisioned with omnivore dung and increased with brood ball weight in offspring provisioned with herbivore dung but not omnivore dung (Figure 5c). However, the effect of food provisioned on horn length was not observed in the minor and major male subsets (Table S2). Testes weight was greater in offspring provisioned with omnivore dung compared to those provisioned with herbivore dung, but was not significantly affected by the amount of dung provisioned nor its interaction with food quality (dung type) (Figure 5d, Table S2).

## 5 Discussion

Differences in reproductive trait expression may occur when males vary their investment in traits according to their ability to bear costs in development or maintenance of the traits (Jennions, Moller, and Petrie 2001). Variation in this ability may be strictly environmental, strictly genetic or via G × E interactions (Jennions, Moller, and Petrie 2001; Wade 2014). Here, we demonstrated that phenotypic variation in sexual traits of *Onthophagus* c.f. *babirussa* was affected by both resource quality and parental provisioning.

### 5.1 Parental Provisioning Varied Based on Larval Food Quality and Brood Sequence

Factors contributing to trait variation in morphology (such as reproductive traits) and/or behaviour are divided into variation due to either environmental conditions or genetic factors (Hunt and Simmons 2000). However, if the quality of the resource provided by parents varies and reflects genetic differences between individuals, there will be a heritable component in the environment, namely, indirect genetic effects (Wolf et al. 1998). Dung beetle parents control the offspring environment by forming brood dung balls and depositing a single egg in each brood ball (Moczek 1998). No further care is provided, and the amount of dung in each brood ball represents the total amount of food available to the offspring (Moczek 1998). In *O. taurus*, Moczek (1998) found from 12 pairs that parent beetles varied the dung amount provisioned according to dung quality, where the amount of dung provisioned (brood ball weight) was significantly higher with low food quality and vice versa. In 40 pairs of *O*. c.f. *babirussa*, we found that the amount of dung provisioned was significantly higher for herbivore dung than omnivore dung for most parental lines (Figure 2a). Herbivore dung balls were on average 6% heavier than omnivore dung balls by the same pairs (SD = 12.3). In addition, more herbivore dung was needed to achieve similar body sizes and horn lengths as offspring reared in omnivore dung. These results suggest that parent beetles in *O*. c.f. *babirussa* can assess resource quality and adjust food provisioning accordingly in compensation for poorer quality.

Dung resources are often patchy and ephemeral in tropical rainforests (Finn 2001; Hanski and Cambefort 1991). Resource limitations likely drive trade‐offs between benefits of current investment in individual offspring quality (dung amount per offspring) and cost of reproduction in the future (number of offspring to be provided for) (Hunt and Simmons 2000; Hunt and Simmons 2002; Gomez and Kölliker 2013). Therefore, the provisioning of the food amount according to food quality observed in *O*. c.f. *babirussa* may help optimise resource allocation by parents in environments with limited dung resources to maximise fitness (Gomez and Kölliker 2013).

### 5.2 Effect of Larval Food Quality and Parental Provisioning Differed for the Reproductive Traits

Our study supports earlier findings that larval food quantity is an important determinant of adult body size, where increased brood ball weight of either resource resulted in larger body sizes (Moczek and Emlen 1999). In addition, the amount of food provisioned significantly differed between the two dung types, where omnivore dung appeared to be of ‘higher quality’ as the body size of offspring raised on omnivore dung were larger those raised on herbivore dung of similar amounts (Figure 3). Notably, based on the breakpoint fitted model for horn length allometry, all major males developed from brood balls constructed from omnivore dung, while all larvae raised in herbivore dung brood balls developed into minor males. This could imply that for this species in particular, the nutrients present only in omnivore dung may be crucial in reaching the switchpoint for development in horn length that decides if a male becomes a major or minor morph.

For horn length, increase in weight of herbivore dung also resulted in longer horns, but increase in weight of omnivore dung had no effect on horn length. This contradicts the trend seen in *O. taurus* where increasing weight of both dung types resulted in greater horn lengths (Moczek 1998). This is perhaps due to the difference in horn development between *O. taurus* and *O*. c.f. *babirussa*. In *O. taurus* males, both the horn length allometric curve and the horn length‐brood ball curves show a sigmoidal relationship, suggesting that *O. taurus* develops horns via a threshold response related to brood ball mass and/or body size, where horns are only produced after exceeding a threshold (Moczek and Emlen 1999; Moczek 1998). In *O*. c.f. *babirussa*, however, both the horn length allometric curve and horn length‐brood ball curves show the ‘traditional conditional expression’—where horn length in males is directly influenced by brood ball weight and dung type. Even though horn length was shown to scale with body size, the factors and mechanisms influencing the development of these two traits may differ. Offspring reared in omnivore dung are significantly larger than offspring reared in herbivore dung given the same amount of food provisioned, but increased weight of omnivore dung did not result in larger horn length. This suggests that dung quality might be a stronger environmental factor than the dung amount in influencing horn length development. There may also be physical limits for horn length expression, beyond which increased provisioning of higher quality dung no longer leads to increases in horn length (Knell, Pomfret, and Tomkins 2004).

Interestingly, when the breakpoint model is applied to the male beetles to split them into minor and major morphs, the food amount did not significantly influence horn length within each morph type, only doing so when all the males were pooled together. This could suggest that the food amount may play a role in influencing whether males fall into either morph category, but not for variation within each category. Futhermore, a recent study on the Dynastinae proposes that horn development is continuous, even in species where they appear to be bimodal (Packard 2021). Further analysis with this assumption on untransformed data may shed further light on the role of food quantity on horn development in this species.

Several studies have investigated the effect of larval food quality and amount of food provisioned on precopulatory traits (Arellano et al. 2015; Chamorro‐Florescano, Favila, and Macías‐Ordóñez 2011; Emlen 1994, 1997; Hunt and Simmons 2000, 2002; Kishi and Nishida 2006; Moczek 1998; Salomão et al. 2019; Snell‐Rood et al. 2016), but fewer studies focus on the effects on postcopulatory traits in *Onthophagus* (Simmons and Kotiaho 2002; House and Simmons 2007). We found that only larval food quality significantly affected testes weight, where given the same amount of dung, offspring reared in omnivore dung had heavier testes than those reared in herbivore dung. This further indicates that omnivore dung is of higher quality than herbivore dung for *O*. c.f. *babirussa*. However, brood ball weight did not significantly affect testes weight, suggesting that dung quality but not quantity is important in determining testes weight.

### 5.3 Resource Quality Had a Greater Effect on Precopulatory Than Postcopulatory Sexual Traits

Dung type significantly affected both sexual traits and body size, but the effects of ‘higher quality’ omnivore dung had greater influence on sexual traits, with the male horn length showing the greatest response to resource quality, followed by testes weight. This implies that sexual trait expression in *O*. c.f. *babirussa* is disproportionately dependent on resource availability, and that the species invests greatly in precopulatory selection. This corroborates previous findings of extreme horn length allometry by Toh et al. (2022), which documented that the allometric slopes for precopulatory traits were steeper than postcopulatory traits across multiple populations of this species. Our results show that horn length responds more positively to better resource quality than testes weight, suggesting a possibility that resource quality during larval development plays an important role in mediating mate acquisition via male–male competition in this species. This also corroborates House and Simmons (2007), who found that relative horn length but not genital traits was condition‐dependent. Future work can expand on this by including more postcopulatory traits, such as genital sclerite measurements and testes dimensions.

### 5.4 Differential Effects of Environment, Genotype and G × E on Trait Expression

The environmental effect of resource quality (dung type) had significant effects on measured traits from both sexes. Genotype (parental line) had significant effects on females (body size) but not males (body size, horn length and testes weight), while the interaction between genotype and environment had significant effects on only male body size and horn length. Overall, our results suggest that GxE interactions play a larger role in male offspring development.

#### 5.4.1 Differential Effects of Environment, Genotype and G × E on Body Size

Phenotypic variation in body size of both males and females was found to be significantly influenced by the environment (resource quality), but the effect of genotype (parental lines) was only significant in females, while G × E was only significant in males. Body sizes of both male and female offspring raised in omnivore dung were larger than those raised in herbivore dung, suggesting that natural variation in larval food quality may be important in explaining variation in body sizes across wild‐caught individuals. This is congruent with the existing studies of *Onthophagus* that display the importance of larval food quality in determining adult body size (Emlen 1997; Moczek 1998).

Previous studies on *Onthophagus* species did not find evidence of heritable variation for body size, suggesting the prevalence of family effects and their interactions with the environment (Buzatto et al. 2015; Garcia‐Gonzalez and Simmons 2011; Simmons and Garcia‐Gonzalez 2011; Simmons et al. 2009; Simmons and Kotiaho 2002). In line with these results, we also found that environment strongly influences adult phenotype, demonstrating that offspring from different parental lines exhibited varying magnitudes of body size plasticity across the different dung types (i.e., G × E). This suggests that there is indeed genetic variation for body size plasticity, which can explain body size variation found in wild populations (Emlen 1997; Moczek 1998). Interestingly, significant G × E interactions were found in male offspring only, which could be due to male‐specific variance in plasticity like sex‐biased gene expression (Stillwell et al. 2010).

#### 5.4.2 Differential Effects of Environment, Genotype and G × E on Horn Length

Dung type significantly affected male horn length, suggesting condition dependence, corroborating previous studies on *Onthophagus* (Emlen 1997; Moczek 1998). Genetic variation relevant to horn length of males also appears as differences in the magnitude of developmental plasticity in horn growth (i.e., G × E), suggesting that there is some genetic variation for plasticity in horn length despite a lack of significant direct effects of genotype. Secondary sexual traits like horns are likely to exhibit greater additive genetic variation (Pomiankowski and Møller 1995). Kotiaho et al. (2003) found that in *O. taurus*, moderate additive genetic variance for horn length and G × E interactions significantly affected male horn length when reared in different dung types, depending on genetic background. The genetic influence on variance in plasticity could be due to similar mechanisms regulating horn length and body size (Emlen and Nijhout 1999).

Interestingly, our results demonstrate that genotype alone has no significant influence on variation in horn length. Instead, the genetic interaction with the environment via factors such as larval resource quality is incredibly important for horn development and thus male mating strategies. Offspring from small‐horned males could exhibit large horns and vice versa, largely due to parental resource provisioning during development.

#### 5.4.3 Differential Effects of Environment, Genotype and G × E on Testes Weight

In sharp contrast to the earlier traits, only environment, but not genotype and G × E, significantly affected testes weight, a postcopulatory trait. The significant effect of environment but not genotype suggests that testes weight has high phenotypic plasticity that is determined more by resource provisioning than genetic variation. Toh et al. (2022) showed that while horn length scales positively with body size in *O*. c.f. *babirussa*, testes size did not exhibit any significant scaling relationship. While it appears that precopulatory sexual selection is prioritised in this species, postcopulatory sexual selection can still be important, especially since female dung beetles are known to have sperm storage organs to contain sperm from multiple males and exhibit cryptic female choice, driving male sperm competition (Favila et al. 2005; McCullough, Buzatto, and Simmons 2018; Simmons and García‐González 2008). In *Onthophagus* beetles, testes size can be important in sperm competition where larger testes contribute to greater fertilisation success (Simmons and García‐González 2008). Theory predicts that traits associated with male–male competition are costly to produce and heritable (Schulte‐Hostedde, Millar, and Hickling 2005), but we did not find this in *O*. c.f. *babirussa*. Interestingly, our results contradict Simmons and Kotiaho (2002), who reported high heritability in testes weight in *O. taurus*. While it is currently difficult to explain this contradiction, it is possible that heritability of testes weight could vary between species of the large genus *Onthophagus*, where testes weight of offspring of different species may be determined via different mechanisms.

We also found that dung type significantly affected testes weight. In insects, as ejaculates are associated with high costs, theory predicts that only males in good condition will produce larger ejaculates (Perry and Rowe 2010; Simmons 2001). Hence, males with larger testes are assumed to transfer more sperm in ejaculates. However, we did not find any significant G × E interaction affecting testes weight in *O*. c.f. *babirussa*, suggesting that there may be no genetic variation in plasticity of testes weight.

### 5.5 Omnivore Dung as a ‘Higher Quality’ Resource Than Herbivore Dung in the Context of *Onthophagus* c.f. *Babirussa* and Its Ecology

All the measured traits showed greater absolute and relative values for *O*. c.f. *babirussa* offspring that were provisioned omnivore dung compared to those provisioned herbivore dung, suggesting that omnivore dung is indeed a higher quality resource for this species. In Singapore, large herbivores have been largely extirpated, so extant dung beetle species are likely to show preference for or have adapted to utilising the dung of omnivorous mammals such as wild pigs (*Sus scrofa*) and long‐tailed macaques (*Macaca fascicularis*) that make up most of the ‘larger’ mammal biomass in local forests. This is further evidenced by the observation that dung of recently reintroduced large herbivores such as Sambar deer (*Rusa unicolor*) takes a much longer time to be removed, as compared to pig and monkey dung (personal observation). During field sampling, we also observed that traps baited with omnivore dung attracted a greater diversity and abundance of beetles than herbivore dung‐baited traps, and this was true for *O*. c.f. *babirussa*. It is likely that dung preference in parent beetles is linked to the effects of dung type on offspring quality, such that parents are attracted to dung that when provisioned would result in the development of better quality offspring.

Interestingly, we also found that the amount of food provisioned differed significantly between initial and subsequent broods for herbivore dung only, where parents provisioned significantly less herbivore dung for the subsequent brood (Figure 4b,c). This further suggests that omnivore dung is of a high quality that does not require adjustments in the amount of dung provisioned. Conversely, in herbivore dung, parents must invest more and construct larger brood balls to maximise fitness. The decrease in the amount of dung provisioned for later herbivore brood balls could also be due to physiological costs in parental care. In other words, females with greater early offspring provisioning may be unable to upkeep the upregulation of food provisioned using herbivore dung later in life (Alonso‐Alvarez and Velando 2012; Gómez and Kölliker 2013).

## 6 Conclusions

Our study demonstrates that resource quality and parental provisioning have a large effect on phenotypic variation of reproductive traits in the dung beetle *Onthophagus* c.f. *babirussa*. Corroborating studies on other *Onthophagus* species, we find that genetic variation in trait expression exists as G × E and that ‘primary’ sexual traits associated with postcopulatory selection are less plastic than ‘secondary’ sexual traits involved in precopulatory mate acquisition. We also document that parents adjust their provisioning behaviour over time, particularly when faced with the extra task of compensating for poor substrate quality. Resource quality and its consequences for offspring reproductive success have important implications for tropical dung beetle ecology and evolution. Climate change, urbanisation and many other factors threaten and affect mammal diversity and suitable dung resource availability for dung beetle species. These could directly influence the evolution of dung beetle mating systems and also have implications for ecosystem functioning and conservation priorities.

## Author Contributions

**Sean Yap:** conceptualization (equal), data curation (supporting), formal analysis (equal), funding acquisition (supporting), investigation (supporting), methodology (equal), project administration (supporting), resources (equal), supervision (equal), visualization (equal), writing – original draft (equal), writing – review and editing (lead). **Kai Xin Toh:** conceptualization (equal), data curation (lead), formal analysis (equal), investigation (lead), methodology (equal), writing – original draft (equal). **Nalini Puniamoorthy:** conceptualization (supporting), formal analysis (supporting), funding acquisition (lead), methodology (supporting), project administration (lead), resources (equal), supervision (equal), writing – review and editing (supporting).

## Conflicts of Interest

The authors declare no conflicts of interest.

## Supporting information


Data S1.

## Data Availability

Raw data and R codes are uploaded to Data Dryad (DOI: https://doi.org/10.5061/dryad.jdfn2z3m8).

## References

[ece370421-bib-0001] Alonso‐Alvarez, C. , and A. Velando . 2012. “Benefits and Costs of Parental Care.” Evolution of Parental Care 40: 61.

[ece370421-bib-0002] Arellano, L. , C. Castillo‐Guevara , C. Huerta , A. Germán‐García , and C. Lara . 2015. “Effect of Using Different Types of Animal Dung for Feeding and Nesting by the Dung Beetle *Onthophagus lecontei* (Coleoptera: Scarabaeinae).” Canadian Journal of Zoology 93, no. 5: 337–343.

[ece370421-bib-0003] Bates, D. , M. Maechler , B. Bolker , and S. Walker . 2015. “lme4: Linear Mixed‐Effects Models Using Eigen and S4. R Package Version 1.1‐7.”

[ece370421-bib-0004] Bonduriansky, R. 2007. “The Evolution of Condition‐Dependent Sexual Dimorphism.” American Naturalist 169, no. 1: 9–19. 10.1086/510214 17206580

[ece370421-bib-0005] Bonduriansky, R. , A. Maklakov , F. Zajitschek , and R. Brooks . 2008. “Sexual Selection, Sexual Conflict and the Evolution of Ageing and Life Span.” Functional Ecology 22: 443–453.

[ece370421-bib-0006] Buzatto, B. A. , J. S. Kotiaho , J. L. Tomkins , and L. W. Simmons . 2015. “Intralocus Tactical Conflict: Genetic Correlations Between Fighters and Sneakers of the Dung Beetle *Onthophagus taurus* .” Journal of Evolutionary Biology 28, no. 3: 730–738. 25736536 10.1111/jeb.12598

[ece370421-bib-0007] Chamorro‐Florescano, I. A. , M. E. Favila , and R. Macías‐Ordóñez . 2011. “Ownership, Size and Reproductive Status Affect the Outcome of Food Ball Contests in a Dung Roller Beetle: When Do Enemies Share?” Evolutionary Ecology 25, no. 2: 277–289.

[ece370421-bib-0008] Cook, D. 1987. “Sexual Selection in Dung Beetles. 1. A Multivariate Study of the Morphological Variation in 2 Species of *Onthophagus* (Scarabaeidae, Onthophagini).” Australian Journal of Zoology 35, no. 2: 123–132.

[ece370421-bib-0009] Dia, M. , T. C. Wehner , and C. Arellano . 2017. “RGxE: An R Program for Genotype × Environment Interaction Analysis.” American Journal of Plant Sciences 8, no. 7: 1672–1698.

[ece370421-bib-0010] Dury, G. J. , A. P. Moczek , and D. B. Schwab . 2020. “Maternal and Larval Niche Construction Interact to Shape Development, Survival, and Population Divergence in the Dung Beetle *Onthophagus taurus* .” Evolution & Development 22, no. 5: 358–369. 33448595 10.1111/ede.12348

[ece370421-bib-0011] Emlen, D. J. 1994. “Environmental Control of Horn Length Dimorphism in the Beetle *Onthophagus acuminatus* (Coleoptera: Scarabaeidae).” Proceedings of the Royal Society of London, Series B: Biological Sciences 256, no. 1346: 131–136.

[ece370421-bib-0012] Emlen, D. J. 1997. “Diet Alters Male Horn Allometry in the Beetle *Onthophagus acuminatus* (Coleoptera: Scarabaeidae).” Proceedings of the Royal Society of London, Series B: Biological Sciences 264, no. 1381: 567–574.

[ece370421-bib-0013] Emlen, D. J. , L. Corley Lavine , and B. Ewen‐Campen . 2007. “On the Origin and Evolutionary Diversification of Beetle Horns.” Proceedings of the National Academy of Sciences 104, no. suppl_1: 8661–8668. 10.1073/pnas.0701209104 PMC1876444 17494751

[ece370421-bib-0014] Emlen, D. J. , J. Marangelo , B. Ball , and C. W. Cunningham . 2005. “Diversity in the Weapons of Sexual Selection: Horn Evolution in the Beetle Genus *Onthophagus* (Coleoptera: Scarabaeidae).” Evolution 59, no. 5: 1060–1084. 16136805

[ece370421-bib-0015] Emlen, D. J. , and H. F. Nijhout . 1999. “Hormonal Control of Male Horn Length Dimorphism in the Dung Beetle *Onthophagus taurus* (Coleoptera: Scarabaeidae).” Journal of Insect Physiology 45, no. 1: 45–53. 12770395 10.1016/s0022-1910(98)00096-1

[ece370421-bib-0016] Esperk, T. , T. Tammaru , S. Nylin , and T. Teder . 2007. “Achieving High Sexual Size Dimorphism in Insects: Females Add Instars.” Ecological Entomology 32, no. 3: 243–256.

[ece370421-bib-0017] Favila, M. E. 1993. “Some Ecological Factors Affecting the Life‐Style of *Canthon cyanellus cyanellus* (Coleoptera Scarabaeidae): An Experimental Approach.” Ethology Ecology & Evolution 5, no. 3: 319–328.

[ece370421-bib-0018] Favila, M. E. , J. Nolasco , I. C. Florescano , and M. Equihua . 2005. “Sperm Competition and Evidence of Sperm Fertilization Patterns in the Carrion Ball‐Roller Beetle *Canthon cyanellus* Cyanellus LeConte (Scarabaeidae: Scarabaeinae).” Behavioral Ecology and Sociobiology 59, no. 1: 38–43.

[ece370421-bib-0019] Finn, J. A. 2001. “Ephemeral Resource Patches as Model Systems for Diversity‐Function Experiments.” Oikos 92, no. 2: 363–366.

[ece370421-bib-0020] Garcia‐Gonzalez, F. , and L. W. Simmons . 2011. “Good Genes and Sexual Selection in Dung Beetles (*Onthophagus taurus*): Genetic Variance in Egg‐To‐Adult and Adult Viability.” PLoS One 6, no. 1: e16233. 10.1371/journal.pone.0016233. 21267411 PMC3022759

[ece370421-bib-0021] Goh, T. G. 2014. “Preliminary Survey of Dung Beetle Diversity in Krau Wildlife Reserve, Pahang, Malayasia.” Journal of Wildlife and Parks 28, no. 13: 11–36.

[ece370421-bib-0022] Gómez, Y. , and M. Kölliker . 2013. “Maternal Care, Mother–Offspring Aggregation and Age‐Dependent Coadaptation in the European Earwig.” Journal of Evolutionary Biology 26, no. 9: 1903–1911. 23937357 10.1111/jeb.12184

[ece370421-bib-0023] Halffter, G. , C. Huerta , and J. Lopez‐Portillo . 1996. “Parental Care and Offspring Survival in *Copris incertus* say, a Sub‐Social Beetle.” Animal Behaviour 52, no. 1: 133–139.

[ece370421-bib-0024] Hanski, I. , and Y. Cambefort , eds. 1991. Dung Beetle Ecology. New Jersey, USA: Princeton University Press.

[ece370421-bib-0025] Hope, R. M. 2013. “Rmisc: Ryan Miscellaneous. R Package Version, 1(5).”

[ece370421-bib-0026] House, C. M. , and L. W. Simmons . 2007. “No Evidence for Condition‐Dependent Expression of Male Genitalia in the Dung Beetle *Onthophagus taurus* .” Journal of Evolutionary Biology 20, no. 4: 1322–1332. 17584227 10.1111/j.1420-9101.2007.01346.x

[ece370421-bib-0027] Hu, Y. , D. M. Linz , E. S. Parker , et al. 2020. “Developmental Bias in Horned Dung Beetles and Its Contributions to Innovation, Adaptation, and Resilience.” Evolution & Development 22, no. 1–2: 165–180. 31475451 10.1111/ede.12310

[ece370421-bib-0028] Hunt, J. , and L. W. Simmons . 2000. “Maternal and Paternal Effects on Offspring Phenotype in the Dung Beetle *Onthophagus taurus* .” Evolution 54, no. 3: 936–941. 10937266 10.1111/j.0014-3820.2000.tb00093.x

[ece370421-bib-0029] Hunt, J. , and L. W. Simmons . 2002. “The Genetics of Maternal Care: Direct and Indirect Genetic Effects on Phenotype in the Dung Beetle *Onthophagus taurus* .” Proceedings of the National Academy of Sciences 99, no. 10: 6828–6832. 10.1073/pnas.092676199 PMC124488 11983863

[ece370421-bib-0030] Hunt, J. , and L. W. Simmons . 2004. “Optimal Maternal Investment in the Dung Beetle *Onthophagus taurus*?” Behavioral Ecology and Sociobiology 55, no. 3: 302–312.

[ece370421-bib-0031] Jennions, M. D. , A. P. Moller , and M. Petrie . 2001. “Sexually Selected Traits and Adult Survival: A Meta‐Analysis.” Quarterly Review of Biology 76, no. 1: 3–36. 11291569 10.1086/393743

[ece370421-bib-0032] Kassambara, A. 2018. “Ggpubr: ‘Ggplot2’ Based Publication Ready Plots.” https://cran.r‐project.org/package=ggpubr.

[ece370421-bib-0033] Kemp, D. J. , and R. L. Rutowski . 2007. “Condition Dependence, Quantitative Genetics, and the Potential Signal Content of Iridescent Ultraviolet Butterfly Coloration.” Evolution 61, no. 1: 168–183. 17300436 10.1111/j.1558-5646.2007.00014.x

[ece370421-bib-0034] Kijimoto, T. , J. Costello , Z. Tang , A. P. Moczek , and J. Andrews . 2009. “EST and Microarray Analysis of Horn Development in *Onthophagus* Beetles.” BMC Genomics 10, no. 1: 1–13. 19878565 10.1186/1471-2164-10-504 PMC2777201

[ece370421-bib-0036] Kishi, S. , and T. Nishida . 2006. “Adjustment of Parental Investment in the Dung Beetle *Onthophagus atripennis* (Col., Scarabaeidae).” Ethology 112, no. 12: 1239–1245.

[ece370421-bib-0037] Knapp, M. , J. Knappová , and T. Miller . 2013. “Measurement of Body Condition in a Common Carabid Beetle, *Poecilus cupreus*: A Comparison of Fresh Weight, Dry Weight, and Fat Content.” Journal of Insect Science 13, no. 1: 1–10. 23879296 10.1673/031.013.0601 PMC3735054

[ece370421-bib-0038] Knell, R. J. , J. C. Pomfret , and J. L. Tomkins . 2004. “The Limits of Elaboration: Curved Allometries Reveal the Constraints on Mandible Size in Stag Beetles.” Proceedings of the Royal Society B: Biological Sciences 271, no. 1538: 523–528. 10.1098/rspb.2003.2641. PMC1691621 15129963

[ece370421-bib-0039] Kotiaho, J. S. , L. W. Simmons , J. Hunt , and J. L. Tomkins . 2003. “Males Influence Maternal Effects That Promote Sexual Selection: A Quantitative Genetic Experiment With Dung Beetles *Onthophagus taurus* .” American Naturalist 161, no. 6: 852–859. 10.1086/375173 12858271

[ece370421-bib-0040] Kotiaho, J. S. , and J. L. Tomkins . 2001. “The Discrimination of Alternative Male Morphologies.” Behavioral Ecology 12, no. 5: 553–557.

[ece370421-bib-0041] Kudavidanage, E. P. , L. Qie , and J. S. H. Lee . 2012. “Linking Biodiversity and Ecosystem Functioning of Dung Beetles in South and Southeast Asian Tropical Rainforests.” Raffles Bulletin of Zoology 25: 133–146.

[ece370421-bib-0042] Kuznetsova, A. , P. B. Brockhoff , and R. H. B. Christensen . 2017. “lmerTest Package: Tests in Linear Mixed Effects Models.” Journal of Statistical Software 82, no. 13: 1–26. 10.18637/jss.v082.i13.

[ece370421-bib-0043] Lorch, P. D. , S. Proulx , L. Rowe , and T. Day . 2003. “Condition‐Dependent Sexual Selection Can Accelerate Adaptation.” Evolutionary Ecology Research 5, no. 6: 867–881.

[ece370421-bib-0044] McCullough, E. L. , B. A. Buzatto , and L. W. Simmons . 2018. “Population Density Mediates the Interaction Between Pre‐and Postmating Sexual Selection.” Evolution 72, no. 4: 893–905. 29455461 10.1111/evo.13455

[ece370421-bib-0045] Miller, C. W. , G. C. McDonald , and A. J. Moore . 2016. “The Tale of the Shrinking Weapon: Seasonal Changes in Nutrition Affect Weapon Size and Sexual Dimorphism, but Not Contemporary Evolution.” Journal of Evolutionary Biology 29, no. 11: 2266–2275. 27468122 10.1111/jeb.12954

[ece370421-bib-0046] Moczek, A. P. 1998. “Horn Polyphenism in the Beetle *Onthophagus taurus*: Larval Diet Quality and Plasticity in Parental Investment Determine Adult Body Size and Male Horn Morphology.” Behavioral Ecology 9, no. 6: 636–641.

[ece370421-bib-0047] Moczek, A. P. , J. Andrews , T. Kijimoto , Y. Yerushalmi , and D. J. Rose . 2007. “Emerging Model Systems in Evo‐Devo: Horned Beetles and the Origins of Diversity.” Evolution & Development 9, no. 4: 323–328. 17651356 10.1111/j.1525-142X.2007.00168.x

[ece370421-bib-0048] Moczek, A. P. , and D. J. Emlen . 1999. “Proximate Determination of Male Horn Dimorphism in the Beetle *Onthophagus taurus* (Coleoptera: Scarabaeidae).” Journal of Evolutionary Biology 12, no. 1: 27–37.

[ece370421-bib-0083] Muggeo, V. M. . 2008. “Segmented: An R Package to Fit Regression Models with Broken‐Line Relationships.” R news 8, no. 1: 20–25.

[ece370421-bib-0049] Oudin, M. J. , R. Bonduriansky , and H. D. Rundle . 2015. “Experimental Evidence of Condition‐Dependent Sexual Dimorphism in the Weakly Dimorphic Antler Fly *Protopiophila litigata* (Diptera: Piophilidae).” Biological Journal of the Linnean Society 116, no. 1: 211–220.

[ece370421-bib-0084] Packard, G. C. 2021. “Is Allometric Variation in the Cephalic Horn on Male Rhinoceros Beetles Discontinuously Dimorphic?” Evolutionary Biology 48: 233–245.

[ece370421-bib-0050] Parzer, H. F. , and A. P. Moczek . 2008. “Rapid Antagonistic Coevolution Between Primary and Secondary Sexual Characters in Horned Beetles.” Evolution: International Journal of Organic Evolution 62, no. 9: 2423–2428. 18616574 10.1111/j.1558-5646.2008.00448.x

[ece370421-bib-0051] Perry, J. C. , and L. Rowe . 2010. “Condition‐Dependent Ejaculate Size and Composition in a Ladybird Beetle.” Proceedings of the Royal Society B: Biological Sciences 277, no. 1700: 3639–3647. 10.1098/rspb.2010.0810 PMC2982242 20573622

[ece370421-bib-0052] Pomiankowski, A. , and A. P. Møller . 1995. “A Resolution of the Lek Paradox.” Proceedings of the Royal Society of London, Series B: Biological Sciences 260, no. 1357: 21–29.

[ece370421-bib-0053] Priawandiputra, W. , Y. Tsuji , K. A. Widayati , and B. Suryobroto . 2020. “Dung Beetle Assemblages in Lowland Forests of Pangandaran Nature Reserve, West Java, Indonesia.” Biodiversitas Journal of Biological Diversity 21, no. 2: 497–504.

[ece370421-bib-0082] R Core Team . 2020. R: A Language and Environment for Statistical Computing. Vienna, Austria: R Foundation for Statistical Computing. https://www.R‐project.org/.

[ece370421-bib-0054] Rodríguez‐Muñoz, R. , A. Bretman , J. Slate , C. A. Walling , and T. Tregenza . 2010. “Natural and Sexual Selection in a Wild Insect Population.” Science 328, no. 5983: 1269–1272. 20522773 10.1126/science.1188102

[ece370421-bib-0055] Rohner, P. T. , and W. U. Blanckenhorn . 2018. “A Comparative Study of the Role of Sex‐Specific Condition Dependence in the Evolution of Sexually Dimorphic Traits.” American Naturalist 192, no. 6: E202–E215. 10.1086/700096 30444660

[ece370421-bib-0056] Rohner, P. T. , T. Teder , T. Esperk , S. Lüpold , and W. U. Blanckenhorn . 2018. “The Evolution of Male‐Biased Sexual Size Dimorphism Is Associated With Increased Body Size Plasticity in Males.” Functional Ecology 32, no. 2: 581–591.

[ece370421-bib-0057] Salomão, R. P. , M. E. Favila , D. González‐Tokman , and I. A. Chamorro‐Florescano . 2019. “Contest Dynamics for Food and Reproductive Resources Are Defined by Health Condition in a Dung Beetle.” Ethology 125, no. 6: 343–350.

[ece370421-bib-0058] Schulte‐Hostedde, A. I. , J. S. Millar , and G. J. Hickling . 2005. “Condition Dependence of Testis Size in Small Mammals.” Evolutionary Ecology Research 7, no. 1: 143–149.

[ece370421-bib-0059] Schwab, D. B. , H. E. Riggs , I. L. Newton , and A. P. Moczek . 2016. “Developmental and Ecological Benefits of the Maternally Transmitted Microbiota in a Dung Beetle.” American Naturalist 188, no. 6: 679–692. 10.1086/688926 27860508

[ece370421-bib-0060] Senécal, S. , J. C. Riva , R. S. O'Connor , F. Hallot , C. Nozais , and F. Vézina . 2021. “Poor Prey Quality Is Compensated by Higher Provisioning Effort in Passerine Birds.” Scientific Reports 11, no. 1: 11182. 34045619 10.1038/s41598-021-90658-w PMC8159977

[ece370421-bib-0061] Servín‐Pastor, M. , R. P. Salomão , F. Caselín‐Cuevas , et al. 2021. “Malnutrition and Parasitism Shape Ecosystem Services Provided by Dung Beetles.” Ecological Indicators 121: 107205.

[ece370421-bib-0062] Silva, D. P. , B. Vilela , B. A. Buzatto , A. P. Moczek , and J. Hortal . 2016. “Contextualized Niche Shifts Upon Independent Invasions by the Dung Beetle *Onthophagus taurus* .” Biological Invasions 18, no. 11: 3137–3148.

[ece370421-bib-0063] Simmons, L. W. 2001. Sperm Competition and Its Evolutionary Consequences in the Insects. 68th ed. Princeton, NJ: Princeton University Press.

[ece370421-bib-0064] Simmons, L. W. 2014. “Sexual Selection and Genital Evolution.” Austral Entomology 53, no. 1: 1–17.

[ece370421-bib-0065] Simmons, L. W. , and D. J. Emlen . 2006. “Evolutionary Trade‐Off Between Weapons and Testes.” Proceedings of the National Academy of Sciences 103, no. 44: 16346–16351. 10.1073/pnas.0603474103 PMC1637585 17053078

[ece370421-bib-0066] Simmons, L. W. , D. J. Emlen , and J. L. Tomkins . 2007. “Sperm Competition Games Between Sneaks and Guards: A Comparative Analysis Using Dimorphic Male Beetles.” Evolution 61, no. 11: 2684–2692. 17941836 10.1111/j.1558-5646.2007.00243.x

[ece370421-bib-0067] Simmons, L. W. , and F. García‐González . 2008. “Evolutionary Reduction in Testes Size and Competitive Fertilization Success in Response to the Experimental Removal of Sexual Selection in Dung Beetles.” Evolution: International Journal of Organic Evolution 62, no. 10: 2580–2591. 18691259 10.1111/j.1558-5646.2008.00479.x

[ece370421-bib-0068] Simmons, L. W. , and F. Garcia‐Gonzalez . 2011. “Experimental Coevolution of Male and Female Genital Morphology.” Nature Communications 2, no. 1: 374. 10.1038/ncomms1379 21730955

[ece370421-bib-0069] Simmons, L. W. , C. M. House , J. Hunt , and F. García‐González . 2009. “Evolutionary Response to Sexual Selection in Male Genital Morphology.” Current Biology 19, no. 17: 1442–1446. 19664925 10.1016/j.cub.2009.06.056

[ece370421-bib-0070] Simmons, L. W. , and J. S. Kotiaho . 2002. “Evolution of Ejaculates: Patterns of Phenotypic and Genotypic Variation and Condition Dependence in Sperm Competition Traits.” Evolution 56, no. 8: 1622–1631. 12353755 10.1111/j.0014-3820.2002.tb01474.x

[ece370421-bib-0071] Simmons, L. W. , and T. J. Ridsdill‐Smith , eds. 2011. Ecology and Evolution of Dung Beetles. River Street Hoboken, NJ: John Wiley & Sons.

[ece370421-bib-0072] Singmann, H. , B. Bolker , and J. Westfall . 2020. “Afex: Analysis of Factorial Experiments.” https://cran.r‐project.org/package=afex.

[ece370421-bib-0073] Snell‐Rood, E. C. , M. Burger , Q. Hutton , and A. P. Moczek . 2016. “Effects of Parental Care on the Accumulation and Release of Cryptic Genetic Variation: Review of Mechanisms and a Case Study of Dung Beetles.” Evolutionary Ecology 30, no. 2: 251–265.

[ece370421-bib-0074] Stillwell, R. C. , W. U. Blanckenhorn , T. Teder , G. Davidowitz , and C. W. Fox . 2010. “Sex Differences in Phenotypic Plasticity Affect Variation in Sexual Size Dimorphism in Insects: From Physiology to Evolution.” Annual Review of Entomology 55: 227–245. 10.1146/annurev-ento-112408-085500 PMC4760685 19728836

[ece370421-bib-0075] Toh, K. X. , S. Yap , T. G. Goh , and N. Puniamoorthy . 2022. “Sexual Size Dimorphism and Male Reproductive Traits Vary Across Populations of a Tropical Rainforest Dung Beetle Species (*Onthophagus babirussa*).” Ecology and Evolution 12, no. 9: e9279. 36177114 10.1002/ece3.9279 PMC9481888

[ece370421-bib-0076] Wade, M. J. 2014. “Genotype‐By‐Environment Interactions and Sexual Selection: Female Choice in a Complex World.” Genotype‐By‐Environment Interactions and Sexual Selection: 1–18. River Street Hoboken, NJ: John Wiley & Sons.

[ece370421-bib-0077] Wickham, H. 2016. “Ggplot2: Elegant Graphics for Data Analysis.”

[ece370421-bib-0078] Wickham, H. 2020. “Tidyr: Tidy Messy Data.” https://cran.r‐project.org/package=tidyr.

[ece370421-bib-0079] Wickham, H. , R. François , L. Henry , and K. Müller . 2020. “Dplyr: A Grammar of Data Manipulation.” https://cran.r‐project.org/package=dplyr.

[ece370421-bib-0080] Wolf, J. B. , E. D. Brodie III , J. M. Cheverud , A. J. Moore , and M. J. Wade . 1998. “Evolutionary Consequences of Indirect Genetic Effects.” Trends in Ecology & Evolution 13, no. 2: 64–69. 21238202 10.1016/s0169-5347(97)01233-0

[ece370421-bib-0081] Zinna, R. , D. Emlen , L. C. Lavine , et al. 2018. “Sexual Dimorphism and Heightened Conditional Expression in a Sexually Selected Weapon in the Asian Rhinoceros Beetle.” Molecular Ecology 27, no. 24: 5049–5072. 30357984 10.1111/mec.14907

