# Supplementary material for: Male Reproductive Traits Display Increased Phenotypic Variation in Response to Resource Quality and Parental Provisioning in a Tropical Rainforest Dung Beetle, Onthophagus c.f. babirussa
Source: Ecol Evol. 2024 Oct 14;14(10):e70421. doi: 10.1002/ece3.70421 (PMC11473793; doi:10.1002/ece3.70421)
Supplement: Supplementary file 1 — Data S1. [file ECE3-14-e70421-s001.docx]

**Supporting Information 1**

Table S1. Countries and coordinates of the sampling sites. Sampling in Singapore was conducted with permission from the National Parks Board, under the research permit number NP/RP18-034-1. The herbivore dung was collected from Gaur cattle in the Singapore Night Safari and the omnivore dung was contributed by members of the study. Contributors had a varied diet consisting red meat, seafood, vegetables and fruit and were not on any medication during the period of contribution. Collected dung of each dung type were thoroughly mixed and stored in -80 freezers. Prior to use in field collection or experiments, dung were thawed to room temperature.

| **Sampling site** | **Coordinates** |
| --- | --- |
| Bukit Batok Nature Park | 1°20'55.2" N, 103°45'49.1" E |
| Chestnut Nature Park | 1°22'31.8" N, 103°46'51.8" E |
| Mandai | 1°24'11.9"N 103°46'34.0"E |
| Upper Seletar Reservoir Park | 1°23'56.8" N, 103°48'20.4” E |
| Windsor Nature Park | 1°21'35.6" N, 103°49'24.9" E |
| Pulau Ubin | 1°24'49.9" N, 103°58'36.0" E |

**Supporting Information 2**

Figure S1. Schematic diagram for husbandry and experimental design.

**Supporting Information 3**

**
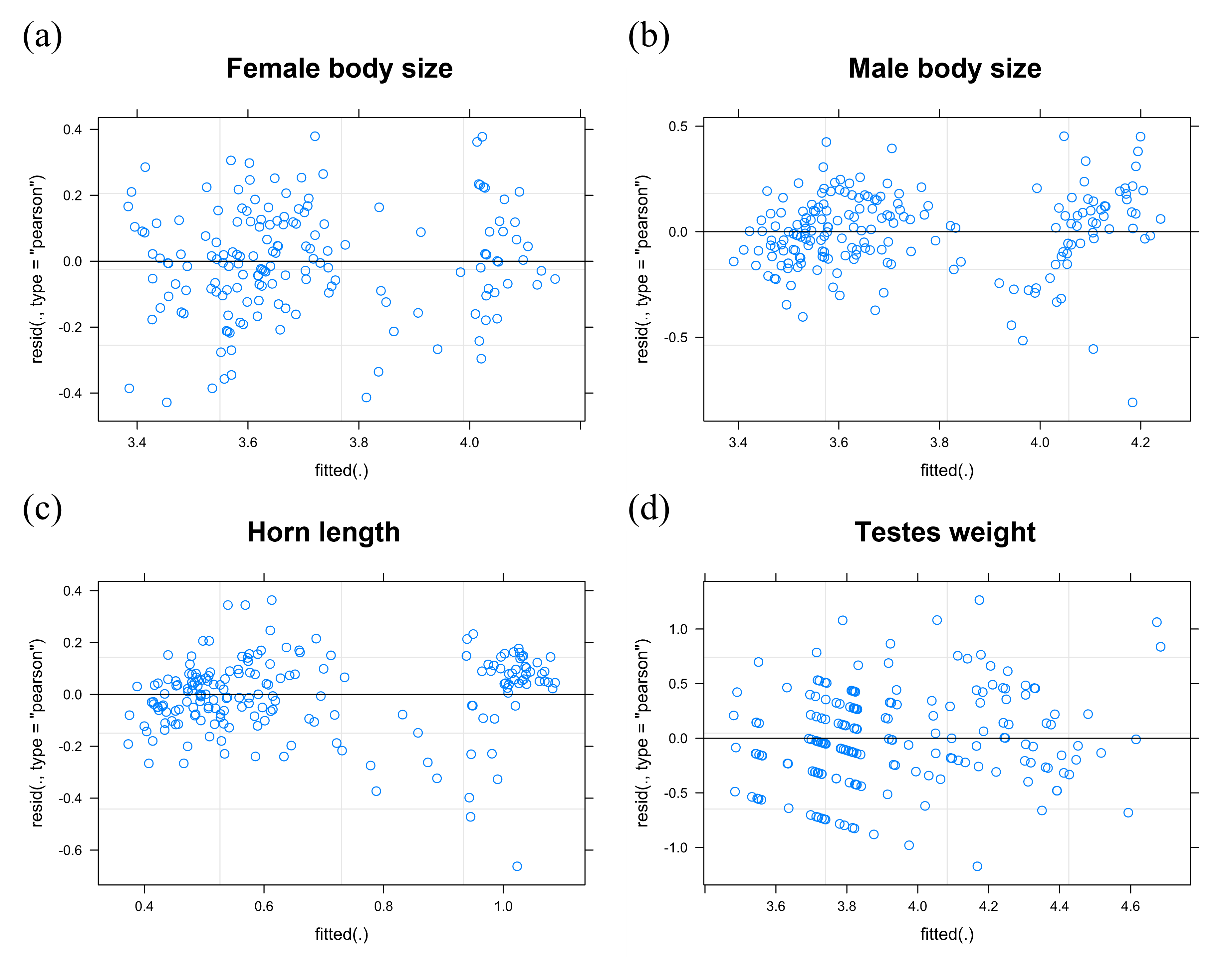
**

Figure S2. Residual plots of the models where lmer(Trait~Dung + BBW + Dung:BBW + (1|Pair) + (1|Pair:Dung). Horn length was square root transformed while testes weight was log transformed to satisfy homoscedasticity assumptions for their models.

**Supporting Information 4**

Table S2. Results from ANOVA of models to investigate the effect of larval food quality (environment: dung type) as a fixed effect and parental lines (genotype) and G×E interactions as random effects, as well as interaction between resource type (dung type) and resource amount (brood ball weight). Fixed effects are presented with F ratios while random effects are presented with likelihood ratios. Analysis includes body size (pronotum width, mm) of female offspring from 20 parental lines (n=163), and body size, horn length (mm) and testes weight (μg) of male offspring of 24 parental lines (overall n=193, minor n=161, major n=32). (*=*P*<0.05, **=*P*<0.01, ***=*P*<0.001, NS=not significant).

| Dung Type (E) | | | | | | |
| --- | --- | --- | --- | --- | --- | --- |
| Source of variance | **Sum of square** | **Mean square** | **Degrees of freedom (numerator)** | **Degrees of freedom (denominator)** | **F-value** | **P-value** |
| Pronotum width (female) | 0.22 | 0.22 | 1 | 74.71 | 7.71 | **  0.0069 |
| Pronotum width (all males) | 0.96 | 0.96 | 1 | 183.44 | 25.29 | ***  1.166e-06 |
| Pronotumwidth (minor males) | 0.40 | 0.40 | 1 | 142.52 | 13.95 | *** 0.0002 |
| Horn length (all males) | 0.82 | 0.82 | 1 | 185.09 | 34.61 | ***  1.848e-08 |
| Horn length (minor males) | 0.34 | 0.34 | 1 | 142.03 | 17.59 | *** 4.791e-05 |
| Testes weight (all males) | 1.20 | 1.20 | 1 | 102.85 | 5.82 | *  0.0175 |
| Testes weight (minor males) | 0.72 | 0.72 | 1 | 98.69 | 3.54 | NS  0.0626 |
| Parental Lines (G) | | | | | | |
| Source of variance | **No. of model parameters** | **log-Likelihood** | **AIC** | **Likelihood ratio test statistic** | **Degrees of freedom** | **P-value (Chi- Square)** |
| Pronotum width (female) | 6 | 34.17 | -56.33 | 4.34 | 1 | *  0.0371 |
| Pronotum width (all males) | 6 | 17.57 | -23.14 | 0.00 | 1 | NS  1 |
| Pronotum width (minor males) | 6 | 35.09 | -58.18 | 0.00 | 1 | NS  1 |
| Pronotum width (major males) | 3 | 12.62 | -19.24 | 0.00 | 1 | NS  0.9685 |
| Horn length (all males) | 6 | 58.68 | -105.36 | 0.00 | 1 | NS  1 |
| Horn length (minor males) | 6 | 59.64 | -107.28 | 0.00 | 1 | NS  1 |
| Horn length (major males) | 3 | 52.13 | -98.26 | 0.48 | 1 | NS  0.4881 |
| Testes weight (all males) | 6 | -140.01 | 292.02 | 2.68 | 1 | NS  0.1012 |
| Testes weight (minor males) | 6 | -115.50 | 243.01 | 0.38 | 1 | NS  0.5372 |
| Testes weight (major males) | 3 | -23.26 | 52.52 | 4.40 | 1 | *  0.0349 |
|  |  |  |  |  |  |  |
| G x E | | | | | | |
| Source of variance | **No. of model parameters** | **log-Likelihood** | **AIC** | **Likelihood ratio test statistic** | **Degrees of freedom** | **P-value (Chi- Square)** |
| Pronotum width (female) | 6 | 36.34 | -60.67 | 0.002 | 1 | NS  0.9645 |
| Pronotum width (all males) | 6 | 15.33 | -18.66 | 4.47 | 1 | * 0.0344 |
| Pronotum width (minor males) | 6 | 32.33 | -52.66 | 5.52 | 1 | *  0.0188 |
| Horn length (all males) | 6 | 54.04 | -96.07 | 9.29 | 1 | **  0.0023 |
| Horn length (minor males) | 6 | 55.33 | -98.67 | 8.60 | 1 | ** 0.0033 |
| Testes weight (all males) | 6 | -138.70 | 289.40 | 0.06 | 1 | NS  0.7972 |
| Testes weight (minor males) | 6 | -115.53 | 243.07 | 0.44 | 1 | NS  0.5051 |
| Brood Ball Weight | | | | | | |
| Source of variance | **Sum of square** | **Mean square** | **Degrees of freedom (numerator)** | **Degrees of freedom (denominator)** | **F-value** | **P-value** |
| Pronotum width (female) | 0.13 | 0.13 | 1 | 87.02 | 4.62 | *  0.0343 |
| Pronotum width (all males) | 0.32 | 0.32 | 1 | 189 | 6.88 | **  0.0094 |
| Pronotum width (minor males) | 0.09 | 0.09 | 1 | 152.12 | 3.34 | NS  0.0695 |
| Pronotum width (major males) | 0.02 | 0.02 | 1 | 22.54 | 1.29 | NS  0.2674 |
| Horn length (all males) | 0.16 | 0.16 | 1 | 37.86 | 29.31 | **  0.0094 |
| Horn length (minor males) | 0.04 | 0.04 | 1 | 152.99 | 2.28 | NS 0.1329 |
| Horn length (major males) | 2.29e-06 | 2.29e-06 | 1 | 27.14 | 0.0018 | NS  0.9667 |
| Testes weight (all males) | 0.29 | 0.29 | 1 | 160.32 | 1.43 | NS  0.2326 |
| Testes weight (minor males) | 0.33 | 0.33 | 1 | 150.05 | 1.63 | NS  0.2032 |
| Testes weight (major males) | 0.07 | 0.07 | 1 | 29.13 | 0.70 | NS  0.408 |
| Dung Type x Brood Ball Weight | | | | | | |
| Source of variance | **Sum of square** | **Mean square** | **Degrees of freedom (numerator)** | **Degrees of freedom (denominator)** | **F-value** | **P-value** |
| Pronotum width (female) | 0.003 | 0.003 | 1 | 67.94 | 0.12 | NS  0.7298 |
| Pronotum width (all males) | 0.1 | 0.1 | 1 | 188.48 | 2.57 | NS  0.1102 |
| Pronotum width (minor males) | 0.12 | 0.12 | 1 | 152.12 | 4.15 | *  0.0432 |
| Horn length (all males) | 0.09 | 0.09 | 1 | 189 | 3.94 | *  0.0483 |
| Horn length (minor males) | 0.09 | 0.09 | 1 | 152.99 | 4.82 | *  0.0295 |
| Testes weight (all males) | 0.23 | 0.23 | 1 | 111.10 | 1.15 | NS  0.2853 |
| Testes weight (minor males) | 0.30 | 0.30 | 1 | 115.22 | 1.49 | NS  0.2235 |

**Supporting Information 5**

Table S3. Table showing the development time and the average values of the reproductive traits of parents (F1) and offspring (F2) raised in different larval dung substrates.

| **Parental (F1)** | | | | | | | | | **Offspring (F2)** | | | | | | | | | | | | | | | | | | | | |
| --- | --- | --- | --- | --- | --- | --- | --- | --- | --- | --- | --- | --- | --- | --- | --- | --- | --- | --- | --- | --- | --- | --- | --- | --- | --- | --- | --- | --- | --- |
| Pair ID | Development Time (days) | | Body Size (mm) | | Lifespan (days) | | Horn length (mm) | Testes weight (ug) | Total # brood balls | Average brood ball weight (g) | | | Total # emerged | Herbivore (Females) | | | Omnivore (Females) | | | Herbivore (Males) | | | | | Omnivore (Males) | | | | |
|  | Female | Male | Female | Male | Female | Male |  |  |  | Total | Herbivore | Omnivore |  | # | Body size (mm) | Dev. Time (days) | # | Body size (mm) | Dev. Time (days) | # | Body size (mm) | Dev. Time (days) | Horn length (mm) | Testes size (ug) | # | Body size (mm) | Dev. Time (days) | Horn length (mm) | Testes size (ug) |
| 1 | 19 | 22 | 3.8 | 3.85 | 89 | 52 | 0.680 | 0.180 | 33 | 2.398 | 1.996 | 2.191 | 17 | 6 | 3.688 ± 0.093 | 25.333 ± 0.816 | 3 | 4.058 ± 0.113 | 24.333 ± 1.528 | 6 | 3.692 ± 0.286 | 26.667 ± 1.966 | 0.394 ± 0.32 | 0.05 ± 0.025 | 2 | 3.587 ± 0.124 | 28 ± 0 | 0.213 ± 0.058 | 0.04 ± 0.014 |
| 2 | 18 | 19 | 3.45 | 3.675 | 119 | 86 | 0.430 | 0.170 | 32 | 1.610 | 1.664 | 1.635 | 23 | 4 | 3.388 ± 0.232 | 28.25 ± 4.717 | 2 | 3.875 ± 0.177 | 25.5 ± 2.121 | 11 | 3.445 ± 0.18 | 25.818 ± 1.722 | 0.264 ± 0.138 | 0.051 ± 0.018 | 6 | 4.029 ± 0.293 | 25.5 ± 2.074 | 1.028 ± 0.349 | 0.075 ± 0.024 |
| 3 | 23 | 23 | 3.95 | 3.45 | 77 | 66 | 0.227 | 0.090 | 26 | 2.135 | 2.121 | 2.128 | 13 | 4 | 3.719 ± 0.142 | 25.5 ± 1.915 | 2 | 4.037 ± 0.159 | 24 ± 2.828 | 6 | 3.767 ± 0.134 | 24.333 ± 1.366 | 0.492 ± 0.18 | 0.062 ± 0.01 | 1 | 4.25 ± 0 | 26 ± 0 | 1.248 ± 0 | 0.05 ± 0 |
| 4 | 20 | 20 | 4.05 | 3.75 | 133 | 76 | 0.485 | 0.190 | 32 | 2.331 | 1.898 | 2.115 | 16 | 6 | 3.763 ± 0.177 | 25.667 ± 1.506 | 1 | 4.15 ± 0 | 27 ± 0 | 5 | 3.69 ± 0.238 | 25.6 ± 0.894 | 0.44 ± 0.268 | 0.066 ± 0.034 | 4 | 3.919 ± 0.246 | 27 ± 4.082 | 0.906 ± 0.47 | 0.078 ± 0.021 |
| 5 | 20 | 22 | 3.75 | 4.075 | 83 | 55 | 1.079 | 0.120 | 27 | 2.027 | 2.005 | 2.016 | 15 | 5 | 3.685 ± 0.152 | 25 ± 1.414 | 2 | 3.788 ± 0.088 | 25.5 ± 2.121 | 6 | 3.638 ± 0.166 | 26.167 ± 1.329 | 0.284 ± 0.168 | 0.063 ± 0.024 | 2 | 4.188 ± 0.053 | 24 ± 0 | 1.21 ± 0.049 | 0.075 ± 0.021 |
| 6 | 20 | 18 | 4.1 | 4.175 | 100 | 94 | 1.273 | 0.040 | 38 | 2.130 | 2.225 | 2.178 | 26 | 6 | 3.529 ± 0.275 | 26.667 ± 1.633 | 1 | 4.375 ± 0 | 26 ± 0 | 13 | 3.6 ± 0.201 | 25.385 ± 1.325 | 0.237 ± 0.152 | 0.048 ± 0.018 | 6 | 4.254 ± 0.441 | 26.5 ± 2.95 | 1.175 ± 0.515 | 0.077 ± 0.033 |
| 7 | 19 | 15 | 4.075 | 3.55 | 72 | NA | 0.193 | 0.170 | 21 | 2.513 | 1.700 | 2.126 | 12 | 3 | 3.717 ± 0.08 | 26.667 ± 3.055 | 1 | 4.1 ± 0 | 24 ± 0 | 7 | 3.782 ± 0.106 | 25.571 ± 1.718 | 0.457 ± 0.194 | 0.046 ± 0.019 | 1 | 4.35 ± 0 | 26 ± 0 | 1.3 ± 0 | 0.05 ± 0 |
| 8 | 17 | 19 | 3.9 | 4.15 | 95 | 71 | 1.287 | 0.100 | 36 | 2.289 | 1.900 | 2.105 | 24 | 12 | 3.725 ± 0.155 | 25.833 ± 1.403 | 3 | 4.117 ± 0.161 | 25 ± 2 | 5 | 3.785 ± 0.176 | 25.4 ± 0.894 | 0.501 ± 0.222 | 0.086 ± 0.048 | 4 | 4.325 ± 0.221 | 24.5 ± 2.38 | 1.314 ± 0.124 | 0.082 ± 0.019 |
| 9 | 22 | 22 | 3.9 | 3.5 | 59 | 55 | 0.210 | 0.050 | 17 | 1.903 | 2.261 | 2.071 | 10 | 4 | 3.55 ± 0.163 | 25.25 ± 1.708 | 0 | NA | NA | 4 | 3.7 ± 0.117 | 25 ± 0.816 | 0.428 ± 0.134 | 0.035 ± 0.013 | 2 | 3.688 ± 0.018 | 28 ± 4.243 | 0.438 ± 0.091 | 0.02 ± 0 |
| 10 | 22 | 15 | 3.5 | 4.25 | 22 | 58 | NA | NA | 0 | NA | NA | NA | 0 | 0 | NA | NA | 0 | NA | NA | 0 | NA | NA | NA | NA | 0 | NA | NA | NA | NA |
| 11 | 23 | 19 | 3.65 | 3.725 | 112 | 52 | 0.509 | 0.120 | 27 | 2.012 | 2.003 | 2.007 | 16 | 4 | 3.744 ± 0.085 | 25 ± 1.414 | 1 | 4.2 ± 0 | 24 ± 0 | 8 | 3.641 ± 0.123 | 26.875 ± 3.907 | 0.32 ± 0.099 | 0.032 ± 0.018 | 3 | 4 ± 0.238 | 25.333 ± 3.512 | 0.97 ± 0.351 | 0.077 ± 0.047 |
| 12 | 22 | 23 | 3.65 | 3.925 | 52 | NA | 0.868 | 0.100 | 13 | 2.093 | 2.086 | 2.091 | 7 | 4 | 3.769 ± 0.069 | 26.25 ± 2.63 | 1 | 4.1 ± 0 | 28 ± 0 | 2 | 3.625 ± 0.247 | 26 ± 0 | 0.364 ± 0.242 | 0.05 ± 0.014 | 0 | NA | NA | NA | NA |
| 13 | 18 | 19 | 3.8 | 4.05 | 112 | 97 | 0.932 | 0.030 | 14 | 1.883 | 1.856 | 1.869 | 8 | 2 | 3.5 ± 0.141 | 27.5 ± 2.121 | 1 | 4.25 ± 0 | 25 ± 0 | 5 | 3.51 ± 0.167 | 28.4 ± 3.286 | 0.264 ± 0.167 | 0.05 ± 0.019 | 0 | NA | NA | NA | NA |
| 14 | 19 | 19 | 4.15 | 4.25 | 117 | 106 | 1.136 | 0.070 | 11 | 2.047 | 1.992 | 2.017 | 5 | 1 | 3.55 ± 0 | 23 ± 0 | 0 | NA | NA | 4 | 3.431 ± 0.085 | 28.25 ± 1.5 | 0.152 ± 0.037 | 0.035 ± 0.017 | 0 | NA | NA | NA | NA |
| 15 | 19 | 18 | 4.025 | 4 | 77 | NA | 1.017 | 0.190 | 15 | 2.008 | 1.911 | 1.956 | 8 | 5 | 3.685 ± 0.156 | 23.6 ± 5.941 | 0 | NA | NA | 2 | 3.612 ± 0.053 | 26 ± 2.828 | 0.225 ± 0.013 | 0.045 ± 0.007 | 1 | 4.25 ± 0 | 21 ± 0 | 1.308 ± 0 | 0.07 ± 0 |
| 16 | 17 | 18 | 4.175 | 4.25 | 98 | NA | 1.179 | 0.190 | 16 | 2.183 | 1.888 | 2.036 | 12 | 5 | 3.6 ± 0.177 | 27.8 ± 1.643 | 2 | 4.088 ± 0.053 | 24.5 ± 0.707 | 3 | 3.525 ± 0.238 | 28.333 ± 1.155 | 0.239 ± 0.068 | 0.04 ± 0.02 | 2 | 4.088 ± 0.124 | 28.5 ± 2.121 | 1.11 ± 0.065 | 0.095 ± 0.049 |
| 17 | 18 | 19 | 3.9 | 4.15 | 133 | 68 | 1.281 | 0.170 | 18 | 1.717 | 2.019 | 1.885 | 10 | 1 | 3.725 ± 0 | 25 ± 0 | 1 | 4.05 ± 0 | 23 ± 0 | 6 | 3.5 ± 0.117 | 26 ± 1.549 | 0.238 ± 0.155 | 0.04 ± 0.014 | 2 | 4.075 ± 0.177 | 23.5 ± 0.707 | 0.851 ± 0.479 | 0.065 ± 0.021 |
| **Parental (F1)** | | | | | | | | | **Offspring (F2)** | | | | | | | | | | | | | | | | | | | | |
| Pair ID | Development Time (days) | | Body Size (mm) | | Lifespan (days) | | Horn length (mm) | Testes weight (ug) | Total # brood balls | Average brood ball weight (g) | | | Total # emerged | Herbivore (Females) | | | Omnivore (Females) | | | Herbivore (Males) | | | | | Omnivore (Males) | | | | |
|  | Female | Male | Female | Male | Female | Male |  |  |  | Total | Herbivore | Omnivore |  | # | Body size (mm) | Dev. Time (days) | # | Body size (mm) | Dev. Time (days) | # | Body size (mm) | Dev. Time (days) | Horn length (mm) | Testes size (ug) | # | Body size (mm) | Dev. Time (days) | Horn length (mm) | Testes size (ug) |
| 18 | 14 | 19 | 3.65 | 3.95 | 13 | 39 | 0.836 | 0.290 | 20 | 1.993 | 1.762 | 1.878 | 13 | 6 | 3.408 ± 0.218 | 26.5 ± 1.871 | 2 | 3.575 ± 0.247 | 26 ± 1.414 | 4 | 3.506 ± 0.105 | 25.75 ± 1.258 | 0.312 ± 0.076 | 0.03 ± 0.008 | 1 | 3.9 ± 0 | 26 ± 0 | 0.765 ± 0 | 0.04 ± 0 |
| 19 | 19 | 24 | 4.025 | 3.95 | 36 | NA | 0.725 | 0.240 | 3 | 1.900 | 2.586 | 2.357 | 1 | 0 | NA | NA | 0 | NA | NA | 1 | 3.8 ± 0 | 24 ± 0 | 0.503 ± 0 | 0.05 ± 0 | 0 | NA | NA | NA | NA |
| 20 | 20 | 19 | 4.15 | 4.275 | 105 | 43 | 1.219 | 0.070 | 36 | 2.275 | 2.205 | 2.240 | 23 | 9 | 3.578 ± 0.281 | 29.778 ± 3.768 | 4 | 3.962 ± 0.205 | 25.75 ± 0.957 | 8 | 3.572 ± 0.292 | 28.25 ± 2.659 | 0.228 ± 0.124 | 0.039 ± 0.011 | 2 | 4.312 ± 0.053 | 27.5 ± 4.95 | 1.179 ± 0.023 | 0.09 ± 0.057 |
| 21 | 20 | 16 | 3.85 | 3.875 | 51 | 45 | 0.834 | 0.110 | 9 | 1.820 | 1.581 | 1.687 | 5 | 1 | 3.4 ± 0 | 22 ± 0 | 0 | NA | NA | 3 | 3.408 ± 0.08 | 29.667 ± 1.528 | 0.2 ± 0.046 | 0.027 ± 0.006 | 1 | 4.25 ± 0 | 24 ± 0 | 1.398 ± 0 | 0.05 ± 0 |
| 22 | 17 | 22 | 3.95 | 3.65 | 54 | 59 | 0.521 | 0.150 | 2 | 1.809 |  | 1.809 | 2 | 2 | 3.6 ± 0 | 26 ± 1.414 | 0 | NA | NA | 0 | NA | NA | NA | NA | 0 | NA | NA | NA | NA |
| 23 | 17 | 17 | 3.375 | 4.2 | 101 | 88 | 1.309 | 0.070 | 15 | 1.608 | 1.390 | 1.492 | 8 | 4 | 3.456 ± 0.12 | 28.25 ± 4.113 | 0 | NA | NA | 2 | 3.45 ± 0.141 | 26.5 ± 0.707 | 0.299 ± 0.076 | 0.075 ± 0.078 | 2 | 4.1 ± 0 | 26 ± 0 | 1.12 ± 0.033 | 0.07 ± 0.042 |
| 24 | 17 | 17 | 4 | 3.775 | 70 | 30 | 0.530 | 0.070 | 24 | 2.186 | 1.860 | 2.023 | 13 | 8 | 3.631 ± 0.142 | 27 ± 0.756 | 1 | 4.05 ± 0 | 23 ± 0 | 3 | 3.55 ± 0.066 | 27 ± 2.646 | 0.285 ± 0.017 | 0.04 ± 0.017 | 1 | 3.925 ± NA | 20 ± NA | 1.096 ± NA | 0.12 ± NA |
| 25 | 19 | 19 | 4.2 | 4.25 | 104 | 137 | 1.205 | 0.140 | 14 | 2.382 | 2.089 | 2.236 | 13 | 1 | 3.85 ± 0 | 23 ± 0 | 2 | 4.175 ± 0.106 | 24.5 ± 2.121 | 6 | 3.712 ± 0.134 | 25.333 ± 1.506 | 0.469 ± 0.256 | 0.098 ± 0.076 | 4 | 4.112 ± 0.392 | 28.25 ± 2.63 | 0.888 ± 0.492 | 0.178 ± 0.123 |
| 26 | 20 | 19 | 4.075 | 4.275 | 31 | 25 | 1.245 | 0.230 | 1 | 1.267 | 1.267 | 1.267 | 1 | 0 | NA | NA | 0 | NA | NA | 0 | NA | NA | NA | NA | 1 | 4.15 ± 0 | 20 ± 0 | 1.184 ± 0 | 0.07 ± 0 |
| 27 | 24 | 24 | 4.05 | 4.15 | 19 | 20 | NA | NA | 0 | NA | NA | NA | 0 | 0 | NA | NA | 0 | NA | NA | 0 | NA | NA | NA | NA | 0 | NA | NA | NA | NA |
| 28 | 24 | 19 | 4.05 | 3.55 | 19 | 19 | 0.448 | 0.140 | 2 | 2.006 | 2.006 | 2.006 | 1 | 0 | NA | NA | 0 | NA | NA | 0 | NA | NA | NA | NA | 1 | 3.45 ± 0 | 35 ± 0 | 0.32 ± 0 | 0.02 ± 0 |
| 29 | 19 | 24 | 4.1 | 4.35 | 24 | 19 | 1.290 | 0.090 | 3 | 1.826 | 1.826 | 1.826 | 0 | 0 | NA | NA | 0 | NA | NA | 0 | NA | NA | NA | NA | 0 | NA | NA | NA | NA |
| 30 | 21 | 19 | 3.8 | 4.275 | 12 | 25 | NA | NA | 0 | NA | NA | NA | 0 | 0 | NA | NA | 0 | NA | NA | 0 | NA | NA | NA | NA | 0 | NA | NA | NA | NA |
| 31 | 17 | 24 | 4.15 | 4.15 | 8 | 20 | NA | NA | 0 | NA | NA | NA | 0 | 0 | NA | NA | 0 | NA | NA | 0 | NA | NA | NA | NA | 0 | NA | NA | NA | NA |
| 32 | 24 | 22 | 4.25 | 4.25 | 82 | 37 | 1.361 | 0.160 | 16 | 1.770 | 1.907 | 1.839 | 15 | 6 | 3.5 ± 0.042 | 26.167 ± 0.753 | 4 | 4.1 ± 0.191 | 25.75 ± 2.062 | 2 | 3.413 ± 0.124 | 27 ± 0 | 0.178 ± 0.093 | 0.035 ± 0.007 | 3 | 4.308 ± 0.101 | 25 ± 1.732 | 1.301 ± 0.042 | 0.11 ± 0.017 |
| 33 | 24 | 19 | NA | 4.175 | NA | NA | NA | NA | 0 | NA | NA | NA | 0 | 0 | NA | NA | 0 | NA | NA | 0 | NA | NA | NA | NA | 0 | NA | NA | NA | NA |
| 34 | 24 | 20 | 4.1 | NA | NA | NA | NA | NA | 0 | NA | NA | NA | 0 | 0 | NA | NA | 0 | NA | NA | 0 | NA | NA | NA | NA | 0 | NA | NA | NA | NA |
| 35 | 24 | 24 | 4.275 | 4.25 | 163 | 70 | 1.143 | 0.150 | 14 | 2.055 | 2.145 | 2.100 | 10 | 0 | NA | NA | 3 | 4.033 ± 0.104 | 24 ± 1 | 6 | 3.483 ± 0.155 | 28.333 ± 3.445 | 0.142 ± 0.084 | 0.042 ± 0.017 | 1 | 4.075 ± 0 | 24 ± 0 | 1.028 ± 0 | 0.08 ± 0 |
| 36 | 19 | 19 | 4.2 | 4.225 | 20 | 20 | NA | NA | 0 | NA | NA | NA | 0 | 0 | NA | NA | 0 | NA | NA | 0 | NA | NA | NA | NA | 0 | NA | NA | NA | NA |
| 37 | 20 | 18 | 4.3 | 4.4 | 51 | 37 | 1.227 | 0.130 | 16 | 2.221 | 1.747 | 2.014 | 12 | 5 | 3.395 ± 0.107 | 28.4 ± 1.949 | 2 | 3.612 ± 0.159 | 25.5 ± 2.121 | 4 | 3.494 ± 0.075 | 25.5 ± 1.291 | 0.213 ± 0.075 | 0.048 ± 0.01 | 1 | 4.1 ± 0 | 23 ± 0 | 1.202 ± 0 | 0.06 ± 0 |
| 38 | 14 | 23 | 4.225 | 4.3 | 62 | NA | 1.398 | 0.100 | 11 | 2.239 | 2.009 | 2.113 | 8 | 4 | 3.55 ± 0.158 | 27.25 ± 1.5 | 1 | 4.4 ± 0 | 26 ± 0 | 1 | 3.325 ± 0 | 26 ± 0 | 0.117 ± 0 | 0.02 ± 0 | 2 | 4.2 ± 0.071 | 25.5 ± 0.707 | 1.313 ± 0.11 | 0.11 ± 0 |
| 39 | 21 | 26 | 4.15 | 4.075 | 40 | 23 | 1.244 | 0.110 | 3 | 1.886 | 1.991 | 1.956 | 1 |  | ± | ± |  | ± | ± | 1 | 3.375 ± 0 | 28 ± 0 | 0.162 ± 0 | 0.06 ± 0 | 0 | NA | NA | NA | NA |
| 40 | 20 | 26 | 4 | 4.35 | 129 | 20 | 1.308 | 0.090 | 11 | 1.471 | 1.612 | 1.535 | 8 | 5 | 3.385 ± 0.206 | 29.8 ± 6.834 |  | ± | ± | 1 | 3.625 ± 0 | 24 ± 0 | 0.305 ± 0 | 0.04 ± 0 | 2 | 4.113 ± 0.124 | 23.5 ± 0.707 | 1.158 ± 0.289 | 0.085 ± 0.035 |
